# Maize leaf yellowing gene *ZmCAAX* modulates growth and drought resistance by regulating abscisic acid contents through interaction with the ABA biosynthetic enzyme ZmNCED3

**DOI:** 10.1111/pbi.70147

**Published:** 2025-06-03

**Authors:** Xiaohu Li, Bin Zhang, Jiyuan Du, Shuai Chen, Yujiao Wang, Qigui Li, Shilin Zhuge, Xinzheng Li, Yongxin Nie, Gaoke Li, Fang Xu, Aiguo Yang, Zhiming Zhang, Haiping Ding

**Affiliations:** ^1^ National Key Laboratory of Wheat Breeding, College of Life Sciences Shandong Agricultural University Taian China; ^2^ Maize Research Institute Sichuan Agricultural University Chengdu China; ^3^ Tobacco Research Institute Chinese Academy of Agricultural Sciences Qingdao China; ^4^ Guangdong Provincial Key Laboratory of Crop Genetic Improvement, Crops Research Institute Guangdong Academy of Agricultural Sciences Guangzhou China; ^5^ The Key Laboratory of Plant Development and Environmental Adaption Biology, Ministry of Education, School of Life Sciences Shandong University Qingdao China

**Keywords:** *Zea mays*, drought stress, abscisic acid, carotenoids, map‐based cloning, ZmCAAX

## Abstract

In maize (*Zea mays* L.), leaves are essential for photosynthesis and transpiration and leaf yellowing is regulated by carotenoid metabolism, hormonal signalling and environmental factors. However, the molecular mechanisms linking drought stress and leaf yellowing remain poorly understood. ZmNCED3, a key regulator of carotenoid degradation and drought stress responses, plays a critical role in ABA biosynthesis, but its upstream regulatory mechanisms remain unclear. This study investigates the association between leaf‐yellowing mutations and drought stress response in maize. Through map‐based cloning and allelism tests, we identified *ZmCAAX* as the causal gene underlying the *yp1* mutant phenotype. *ZmCAAX* encodes a CAAX amino‐terminal protease family protein. Overexpression of *ZmCAAX* increases drought sensitivity, whereas knockout mutants exhibit enhanced drought resistance. ZmCAAX physically interacts with ZmNCED3 and promotes its degradation. Under drought stress, the expression of *ZmCAAX* decreases, resulting in increased *ZmNCED3* levels, which in turn promotes carotenoid degradation and ABA biosynthesis. Based on these findings, designing *ZmCAAX* gene knockouts or selecting natural variant alleles of *ZmCAAX* could significantly enhance drought stress resistance and carotenoid content. This genetic strategy may be applied to maize breeding to improve maize quality and drought stress resistance.

## Introduction

Leaves are crucial vegetative organs in plants, playing a central role in synthesizing organic matter through photosynthesis and regulating transpiration (Leister, 2023; Wang *et al*., 2020a). Leaf pigmentation changes dynamically throughout the plant life cycle, transitioning from pale yellow to green and later from green to brown or yellow (Guo *et al*., 2021). These colour transitions are closely associated with dynamic fluctuations in pigments, such as chlorophyll and carotenoids, which play a crucial role in leaf growth and development (Jiang *et al*., 2023; Wang *et al*., 2021a). Leaf colour mutants can be classified into five types based on pigmentation: albino, yellow, light green, striped and spotted (Huang *et al*., 2024; Li *et al*., 2021a; Zhang *et al*., 2024). Despite the broad classification of leaf colour mutants, the molecular mechanisms underlying yellow‐leaf mutations remain poorly understood. In *Arabidopsis*, the *senescence‐associated ubiquitin ligase 1* (*SAUL1*) gene encodes an E3 ubiquitin ligase and mutations in this gene lead to leaf yellowing and decreased chlorophyll levels (Drechsel *et al*., 2011; Salt *et al*., 2011; Tong *et al*., 2017). Additionally, *jumonji domain‐containing 17* (*JMJ17*), an H3K4me3 demethylase, regulates cotyledon greening during the seedling stage in *Arabidopsis*, playing a pivotal role in leaf pigmentation control (Huang *et al*., 2019; Islam *et al*., 2021; Wang *et al*., 2021b). In maize, the leaf colour mutant *etiolated/albino leaf 1* (*eal1*) exhibits yellow or albino leaves due to a mutation in the *ZmSig2A* gene, which encodes a sigma factor essential for chloroplast transcription. However, studies of yellow‐leaf mutations in maize are still limited (Li *et al*., 2021a).

Leaf yellowing in plants primarily results from processes such as chloroplast development, chlorophyll metabolism (synthesis and degradation) and the regulation of photosynthetic pigment composition (Frangedakis *et al*., 2024; Ling *et al*., 2021; Ye *et al*., 2024). Additionally, under stress conditions, phytohormones, particularly abscisic acid (ABA), play a critical role in modulating chlorophyll degradation, thereby accelerating leaf yellowing (Wei *et al*., 2022). Drought stress disrupts chlorophyll biosynthesis and alters carotenoid balance, which contributes to leaf yellowing (Gupta *et al*., 2020; Munne‐Bosch and Villadangos, 2023; Zhu, 2016). Moreover, the conversion of chloroplasts into chromoplasts has been identified as another key mechanism influencing leaf yellowing under stress conditions (Ling *et al*., 2021; Llorente *et al*., 2020; Martinez *et al*., 2024). These leaf colour changes reflect a dynamic balance between chlorophyll and carotenoids, regulated by both environmental cues and developmental signals (Zhu *et al*., 2023). Although the metabolism of chlorophyll and carotenoids has been explored in various studies, the molecular mechanisms underlying their coregulation or cross‐regulation remain to be fully understood.

Drought stress significantly affects plant growth and development; the trade‐off between growth, development and drought resistance is a common phenomenon. However, the molecular mechanisms linking leaf colour regulation and drought stress remain largely unknown (Gao *et al*., 2022; Wang *et al*., 2016). Recent studies suggest that the maize gene *ZmSRO1d* enhances reactive oxygen species (ROS) accumulation in guard cells, thereby promoting stomatal closure, a critical mechanism for balancing maize yield and drought resistance (Gao *et al*., 2022). In *Brassica napus*, the transcription factor *BnaABF3* plays a pivotal role in regulating leaf yellowing and drought response by directly binding to the *BnaZEPs* promoter, thereby enhancing carotenoid and ABA biosynthesis (Ye *et al*., 2024). Furthermore, β‐carotene hydroxylase (BCH) modulates carotenoid levels in *Daucus carota*, thereby influencing drought resistance and leaf colour (Li *et al*., 2021b). Carotenoids, as essential precursors of phytohormones, not only determine leaf colour but also play a crucial role in ABA biosynthesis and drought resistance (Chen *et al*., 2024a; Sun *et al*., 2024).

Carotenoids are biosynthesized from geranylgeranyl diphosphate (GGPP), a precursor derived from the methylerythritol phosphate (MEP) pathway (Chen *et al*., 2024b; Ye *et al*., 2024). Carotenoids function as precursors for ABA biosynthesis, with zeaxanthin epoxidase (ZEP) catalysing the conversion of zeaxanthin to violaxanthin, followed by cleavage by 9‐cis‐epoxycarotenoid dioxygenase (NCED) to produce xanthoxin, the direct precursor of ABA (Chen *et al*., 2023a; Kim *et al*., 2024; Nie *et al*., 2024; Ye *et al*., 2024). The phytohormone ABA serves as a critical regulator of plant growth, development and responses to both biotic and abiotic stress (Daszkowska‐Golec, 2022; Waadt *et al*., 2022). In plants, ZmNCED3 plays a central role in ABA biosynthesis, which is essential for plant growth and development (Sato *et al*., 2018; Seo and Koshiba, 2002). In rice (*Oryza sativa* L.), *OsNCED3* is induced by drought stress but declines during recovery, mirroring fluctuations in ABA levels, while its overexpression enhances salt resistance (Chen *et al*., 2023c; Jiang *et al*., 2024; Xie *et al*., 2024). In *Arabidopsis*, the transcription factor *NGATHA1* (*NGA1*) binds to a 5′ UTR cis‐element of *NCED3*, thereby inducing its drought‐responsive expression (Sato *et al*., 2018). However, the regulatory network governing *ZmNCED3* in maize response to drought stress remains poorly understood, since its upstream regulatory factors remain unknown.

CAAX motifs (where C represents cysteine, A denotes an aliphatic amino acid and X signifies a terminal amino acid) are tetrapeptide sequences involved in protein isoprenylation, a form of post‐translational modification (Reimann *et al*., 2023; Wang *et al*., 2018, 2020b). This C‐terminal modification plays a crucial role in regulating cellular processes, including protein localization, protein–protein interactions and protein stability (Horste *et al*., 2023; Zou *et al*., 2024). In animals, the E3 ubiquitin ligase FBXL2, which contains a CAAX motif, targets the epidermal growth factor receptor (EGFR) protein for degradation and plays a role in disease prevention and treatment (Niu *et al*., 2021). In *Arabidopsis*, the CAAX amino‐terminal protease balance of chlorophyll metabolism (BCM) delays chlorophyll degradation by destabilizing the stay‐green 1 (SGR1) protein (Wang *et al*., 2020c). However, the molecular mechanisms by which CAAX proteins function under drought stress in plants remain unexplored.

This study identifies a maize leaf colour mutant *yellow plant 1* (*yp1*). The leaf‐yellowing phenotype of the *yp1* mutants results from a mutation in *ZmCAAX*. The ZmCAAX protein interacts with ZmNCED3 and promotes its degradation, thereby regulating carotenoid decomposition and ABA biosynthesis *in vivo*. This research marks the first identification of the upstream regulatory factors of ZmNCED3 and establishes a link between leaf yellowing and drought stress, providing a theoretical foundation for balancing plant growth and development with stress resistance. Targeted manipulation of *ZmCAAX* expression may enhance carotenoid accumulation in maize kernels or improve drought resistance, presenting a potential strategy for crop improvement.

## Results

### The *yp1* mutant in maize exhibits leaf yellowing and chloroplast abnormalities

**Figure 1:**
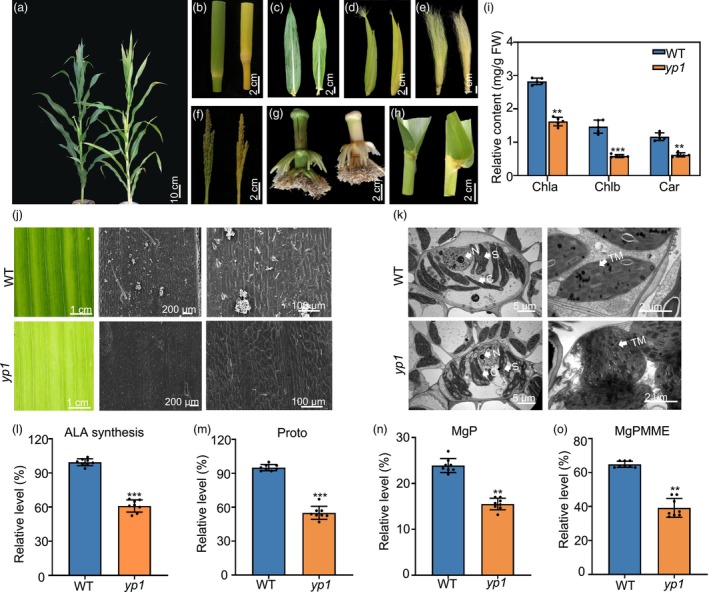
Phenotypic characterization of the maize *yp1* mutants. (a–h) Phenotypic differences between wild‐type (RP125) and *yp1* mutants. (i) Determination of chlorophyll a (Chl a), chlorophyll b (Chl b) and carotenoid (Car) content in wild‐type and *yp1* mutants. Data are means ± SD of three biological replicates. Asterisks indicate significant differences, **P < 0.01, ***P < 0.001, as determined by a two‐sided Student's *t*‐test. (j) Scanning electron microscopy (SEM) observation of epidermal hair and stomatal phenotypes on mature leaves of wild‐type and *yp1* mutant maize. (k) Transmission electron micrographs showing chloroplast structures in wild‐type and *yp1* mutants. C, chloroplast; N, nucleus; S, starch granule; TM, thylakoid membrane. Scale bars: left, 5 μm; right, 2 μm. (l–o) Relative 5‐aminolevulinic acid (ALA) synthesis rate (l), protoporphyrin IX (Proto) (m), Mg‐Proto (MgP) (n) and MgP monomethylester (MgP‐MME) levels (o) in wild‐type and *yp1* leaves grown for 24 days after germination under the same conditions. Data are means ± SD of three biological replicates. Asterisks indicate significant differences, **P < 0.01, ***P < 0.001, as determined by a two‐sided Student's *t*‐test.

A mutant that exhibited significantly yellower leaves was identified in the M_2_ population derived from ethyl methanesulfonate (EMS) mutagenesis of the Chinese elite maize inbred line RP125 (Nie *et al*., 2021). This mutant exhibited normal growth during the seedling stage but progressively developed a distinct leaf‐yellowing phenotype. Thus, we named this mutant ‘*yellow plant 1*’ (*yp1*). One week after germination, *yp1* mutants exhibited growth similar to the wild‐type plants. However, approximately 2 weeks after germination, the stems began to turn yellow and at later stages, the plants developed yellow stems, tassels and female ears (Figure 1a–h; Figure S1a). Chlorophyll a (Chl a), chlorophyll b (Chl b) and carotenoid (Car) content were measured in the second leaves. At 7 days after sowing (DAS), there were no significant differences in these pigments between *yp1* and wild‐type plants. However, by 14 DAS, Chl a, Chl b and carotenoid levels were significantly lower in *yp1* mutants compared to wild‐type plants, with reductions of 27.3%, 17.4% and 33.8%, respectively, by 24 DAS (Figure 1i; Figure S1b). Although plant height did not differ significantly between wild‐type and *yp1* mutants (Figure S1c), the time from sowing to flowering was significantly longer in *yp1* mutants (72.4 ± 1.8 days), than in wild‐type plants (68.3 ± 1.7 days; Figure S1d).

To further investigate the *yp1* leaf phenotype, we performed scanning electron microscopy (SEM) analysis. The *yp1* mutants exhibited completely glabrous leaf blades. SEM analysis revealed the absence of all three types of epidermal hairs, namely macro hairs, prickle hairs and bicellular hairs, on the leaf surface, which appeared smooth (Figure 1j; Figure S2a). To further examine chloroplast development in *yp1* mutants, we used transmission electron microscopy (TEM). Under constant light conditions, *yp1* mutants exhibited significant differences in chloroplast ultrastructure compared with wild‐type plants. In *yp1* mesophyll cells, both the number and overall volume of chloroplasts in the mesophyll cells were markedly reduced (Figure 1k). Moreover, some chloroplasts exhibited disrupted structural integrity and contained enlarged starch granules, although other chloroplasts retained a normal lamellar structure and typical starch granules (Figure 1k).

Immunoblotting and quantitative reverse transcription‐PCR (qRT‐PCR) analyses confirmed protein deficiencies in *yp1* mutants, with significant reductions observed in stromal proteins (RbcL) and thylakoid membrane proteins (PsbD, AtpB) both encoded by the chloroplast genome (Figure S2b,c). To assess whether the abnormal chloroplast development in *yp1* mutants affected chloroplast function, we evaluated 5‐aminolevulinic acid (ALA) synthesis. ALA synthesis was reduced in *yp1* mutants, resulting in slightly lower protoporphyrin IX (Proto) accumulation and decreased magnesium porphyrins, including MgP and MgP monomethylester (MgPMME), which led to reduced chlorophyll contents (Figure 1l–o). These findings suggest that chloroplast development was impaired and chlorophyll synthesis was disrupted in the *yp1* mutants.

### Genetic analysis and map‐based cloning of *ZmCAAX*

**Figure 2:**
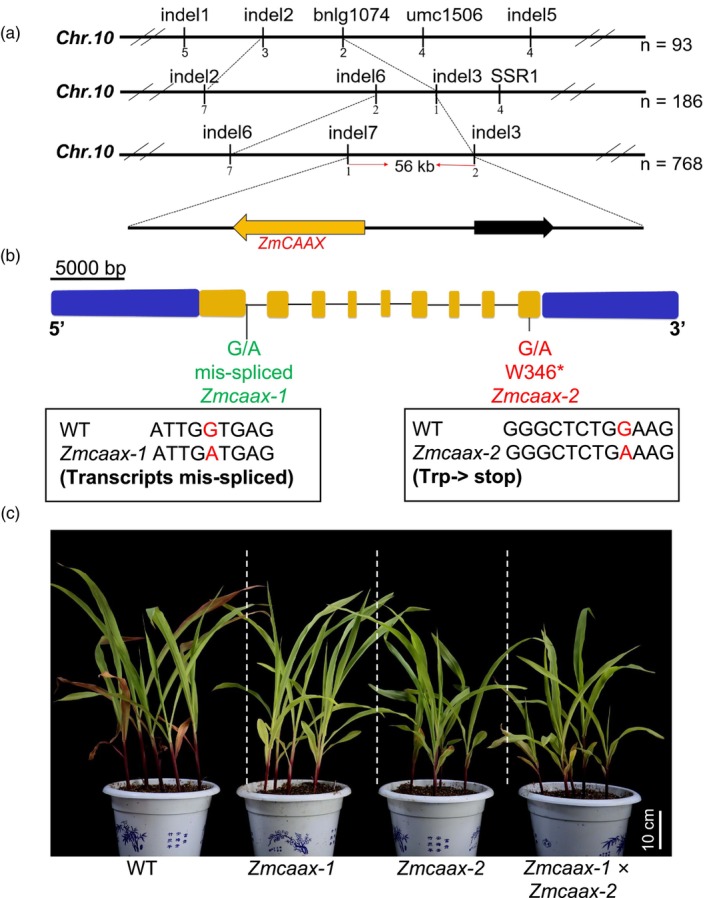
Map‐based cloning and genetic verification of *ZmCAAX*. (a) Positional cloning scheme of *ZmCAAX*. (b) Structural analysis of the gene *ZmCAAX* and identification of mutation sites. Asterisks (*) indicate stop codons. (c) Comparison of leaf colour phenotypes between allelic mutants, their progenies and the wild‐type plants. Scale bars = 10 cm.

To identify and clone the causal gene, we generated a recombinant F_2_ population by crossing recessive *yp1* mutants with the B73 inbred line and performed bulked segregant analysis (BSA) using pooled RNA samples from the leaves of ~40 mutant and wild‐type plants (Gallavotti and Whipple, 2015). The candidate gene was mapped to an ~2 Mb region on the short arm of chromosome 10. By analysing approximately 768 individual mutants, we further narrowed this region to ~56 kb, which contains two candidate genes in the B73 reference genome v4: *Zm00001d025859* (*Zmumc2785*) and *Zm00001d025860* (hereafter referred to as *ZmCAAX*) (Figure 2a; Figure S3a–d).

We then compared the DNA sequences within this interval between wild‐type and *yp1* mutants (Figure 2b; Figure S3e). Sequencing revealed a single nucleotide polymorphism (SNP) transition from G to A at the splice junction of the first exon and the first intron of *Zm00001d025860* (Figure 2b; Figure S3f). This mutation leads to first intron retention, resulting in premature translation termination. Additionally, we identified another mutant in our EMS collection with a similar phenotype, designated as *Zmcaax‐2*. Sequencing of the *ZmCAAX* locus in *Zmcaax‐2* revealed a G‐to‐A mutation in exon 9 of *Zm00001d025860*. This mutation likely changes the tryptophan residue at position 347 into a premature stop codon (Figure 2b; Figure S3g). To confirm allelism, we crossed *yp1* and *Zmcaax‐2* mutants and the resulting F_1_ plants exhibited the same yellow‐leaf phenotype as the parental mutants (Figure 2c; Figure S3h–j), confirming that the two mutants are allelic and that mutations in *Zm00001d025860* produce the yellow‐leaf phenotype.

### Molecular characterization of *ZmCAAX*

**Figure 3:**
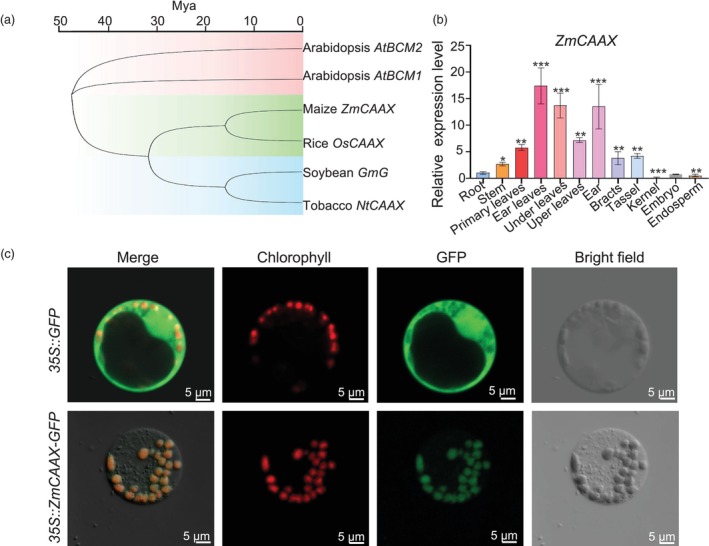
Phylogenetic relationships, expression patterns and subcellular localization of ZmCAAX. (a) Phylogenetic analysis of *ZmCAAX* homologous genes from maize (*Zea mays*), rice (*Oryza sativa*), soybean (*Glycine max*), *Arabidopsis thaliana* and tobacco (*Nicotiana benthamiana*). (b) Expression levels of *ZmCAAX* in different tissues. Data are means ± SD of three biological replicates. Asterisks indicate significant differences: *P < 0.05, **P < 0.01, ***P < 0.001, as determined by a two‐sided Student's *t*‐test. (c) Subcellular localization of the 35S::ZmCAAX‐GFP fusion protein in maize protoplast cells. The fluorescence of 35S::ZmCAAX‐GFP coincides with chlorophyll autofluorescence, confirming chloroplast targeting. In the control, GFP accumulates in the cytosol and nucleus. Scale bars = 5 μm.

The *ZmCAAX* gene spans 2873 bp and comprises nine exons and eight introns. It encodes a protein of approximately 49.84 kDa, consisting of 375 amino acids, featuring a CAAX domain and two transmembrane domains. Multiple sequence alignment revealed that *ZmCAAX* encodes a CAAX amino‐terminal protease family protein. Phylogenetic analysis indicated high homology between the *ZmCAAX*‐encoded protein and orthologs in *Oryza sativa*, *Nicotiana tabacum*, *Glycine max* and *Arabidopsis thaliana* (Figure 3a). *ZmCAAX* is an ortholog of the soybean (*Glycine max* L.) stay‐green *G* gene, which controls seed dormancy and its role is conserved across soybean, rice and *Arabidopsis* (Wang *et al*., 2018). To investigate whether *ZmCAAX* affects seed germination in maize, we measured the germination rate of the *Zmcaax‐1* mutant. Seven days after germination, the germination rate of *Zmcaax‐1* was 23% lower than that of wild‐type plants (Figure S4). To investigate the expression pattern of *ZmCAAX*, we performed qRT‐PCR on various maize tissues including roots, stems, leaves, bracts, ears, tassels and kernels. *ZmCAAX* exhibited the highest expression in leaves, particularly in those with elevated chlorophyll levels (Figure 3b). To determine the subcellular localization of ZmCAAX, GFP and the ZmCAAX‐GFP fusion protein constructs were transiently expressed in maize protoplasts. While GFP alone exhibited uniform fluorescence across the cytoplasm and nucleus, the ZmCAAX‐GFP fusion protein was specifically localized to chloroplasts (Figure 3c). These results indicate that ZmCAAX is localized in chloroplasts and is highly expressed in leaves, consistent with its proposed role in regulating leaf pigmentation by influencing chlorophyll biosynthesis.

### *ZmCAAX* regulates the expression of ABA biosynthetic genes

**Figure 4:**
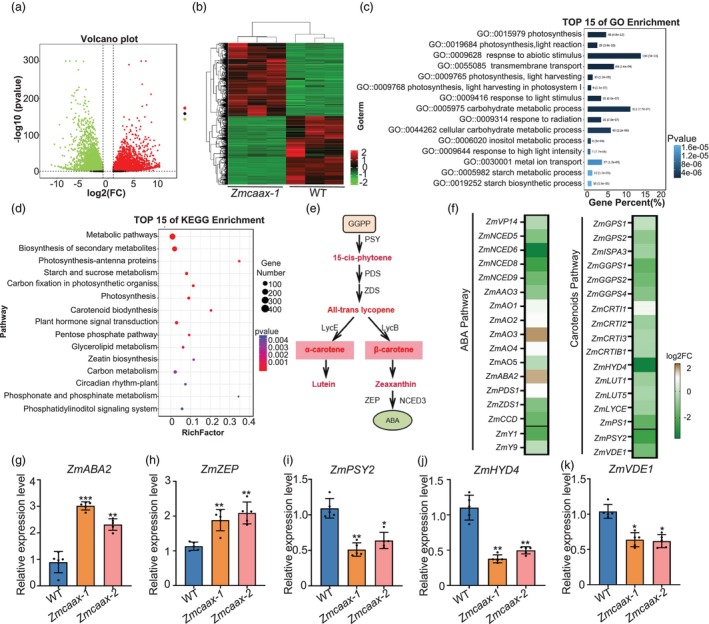
Transcriptome analysis of wild‐type and *Zmcaax* mutants. (a) Volcano plots showing differentially expressed genes (DEGs) between wild‐type and *Zmcaax* mutants. Red dots represent upregulated genes, and green dots represent downregulated genes. Significance thresholds are indicated by dashed lines (log_2_(FC) > 1, *P* < 0.05). (b) Hierarchical clustering of DEGs across three biological replicates. Clustering was performed using average linkage and Euclidean distance metrics. (c) Gene Ontology (GO) functional enrichment analysis. Numbers indicate the number and proportion of enriched genes. (d) Kyoto Encyclopedia of Genes and Genomes (KEGG) pathway enrichment of DEGs. (e) Simplified carotenoid metabolic pathway highlighting genes involved in ABA biosynthesis. Key intermediates and enzymatic steps are labelled. (f) Comparison of expression levels of genes involved in carotenoid and ABA synthesis pathways between wild‐type and *Zmcaax* mutants. (g–k) qRT‐PCR validation of *ZmABA2* (g), *ZmZEP* (h), *ZmPSY2* (i), *ZmHYD4* (j) and *ZmVDE1* (k). Data are means ± SD of three biological replicates. Asterisks indicate significant differences, *P < 0.05, **P < 0.01, ***P < 0.001, as determined by a two‐sided Student's *t*‐test.

To investigate transcriptomic changes in *Zmcaax*‐mutant leaves, RNA‐seq was performed on both wild‐type and *Zmcaax‐1* mutant leaves. Principal component analysis (PCA) revealed a strong correlation among biological replicates for each genotype, confirming the reliability of the RNA‐seq data (Figure S5a). A total of 1070 differentially expressed genes (DEGs) (fold‐change ≥ 1, *P* ≤ 0.05) were identified, including 745 downregulated and 325 upregulated genes in the *Zmcaax‐1* mutants (Table S1; Figure 4a). Hierarchical clustering analysis of these DEGs revealed that many genes with decreased expression in the wild‐type plants exhibited higher expression levels in *Zmcaax‐1* mutants (Figure 4b). Gene ontology (GO) and Kyoto Encyclopedia of Genes and Genomes (KEGG) analyses indicated the involvement of these DEGs in *Zmcaax‐1* mutants in various biological processes, including transmembrane transport, carbohydrate metabolism, photosynthesis, light reactions and responses to abiotic stimuli (Figure 4c,d; Figure S5b,c). These findings suggest that *ZmCAAX* plays a key role in regulating photosynthesis and responses to abiotic stress.

Further transcriptomic analysis revealed that the expression of genes in the methylerythritol phosphate (MEP) and mevalonate (MVA) pathways remained largely unchanged in *Zmcaax‐1* mutants compared to wild‐type plants. However, genes involved in the carotenoid biosynthesis pathway exhibited slight downregulation, whereas those associated with ABA biosynthesis showed an upregulation trend (Figure 4e,f; Figure S5d). To validate the RNA‐seq results, we performed qRT‐PCR to quantify the expression levels of selected DEGs. Consistent with the RNA‐seq data, ABA biosynthesis‐related genes, including *ZmABA2* and *ZmZEP*, were upregulated in the *Zmcaax* mutant lines compared with wild‐type seedlings (Figure 4g,h). Conversely, the expression of carotenoid biosynthesis genes *ZmPSY2*, *ZmHYD4* and *ZmVDE1* was downregulated in the *Zmcaax* mutants (Figure 4i–k). Hormone assays confirmed that ABA levels were elevated in *Zmcaax* mutants (Figure S5e). These findings indicate that *ZmCAAX* plays a crucial role in ABA biosynthesis and contributes to light signal and abiotic stress responses by regulating the expression of ABA‐related genes.

### *ZmCAAX* negatively regulates drought resistance in maize

**Figure 5:**
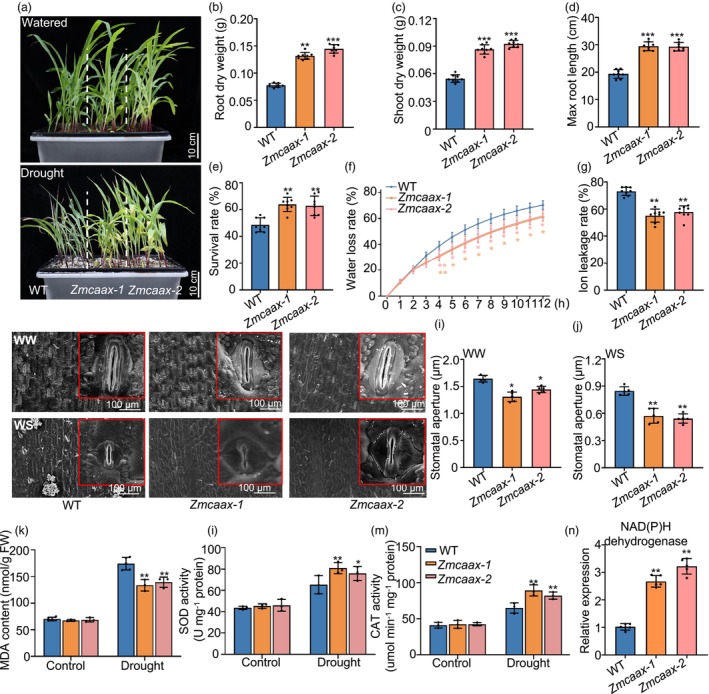
*Zmcaax* mutants improve maize drought resistance. (a) Wild‐type and *Zmcaax*‐mutant plants were grown in pots for 10 days post‐germination and subjected to drought stress (300 mM mannitol) or well‐watered conditions for an additional 2 weeks. Representative images are shown. Scale bars = 10 cm. (b–d) Quantitative description of phenotypes, including root dry weight (b), shoot dry weight (c), max root length (d), under drought stress. Data are means ± SD of three biological replicates. Asterisks indicate significant differences: **P < 0.01, ***P < 0.001, as determined by a two‐sided Student's *t*‐test. (e–g) Survival rate (e), water loss rate (f) and ion leakage rate (g) of wild‐type and *Zmcaax*‐mutant plants after drought stress. Data are means ± SD of three biological replicates. Asterisks indicate significant differences: *P < 0.05, **P < 0.01, ***P < 0.001, as determined by a two‐sided Student's *t*‐test. (h) Representative images of stomatal opening and closing in wild‐type and *Zmcaax* mutants under well‐watered (WW) and drought‐stress (DS) conditions. Scale bar = 100 μm. (i, j) Quantification of stomatal aperture under WW and DS conditions. Measurements were taken from 100 stomata across five replicates. Data are means ± SD of three biological replicates. Asterisks indicate significant differences: *P < 0.05, **P < 0.01, as determined by a two‐sided Student's *t*‐test. (k–m) Determination of malondialdehyde (MDA) contents (k), superoxide dismutase (SOD) activities (l) and catalase (CAT) activities (m) in wild‐type and *Zmcaax* mutants before and after drought treatment. Data are means ± SD of three biological replicates. Asterisks indicate significant differences: *P < 0.05, **P < 0.01, ***P < 0.001, as determined by a two‐sided Student's *t*‐test. (n) qRT‐PCR analysis of NAD(P)H dehydrogenase gene expression levels in wild‐type and *Zmcaax* mutants. Data are means ± SD of three biological replicates. Asterisks indicate significant differences, **P < 0.01, as determined by a two‐sided Student's *t*‐test.

Transcriptome analysis revealed significant alterations in the expression of stress‐responsive genes and ABA biosynthesis‐related genes in the *Zmcaax* mutants (Figure 4c). ABA treatment strongly suppressed *ZmCAAX* expression, as confirmed by transcript level analysis (Figure S6a). Since ABA accumulates in response to drought stress and plays a critical role in drought resistance (Chen *et al*., 2022; Gupta *et al*., 2020; Hu *et al*., 2024), we further examined the response of *ZmCAAX* under drought conditions. Following treatment with 300 mM mannitol (a drought‐mimicking agent), the expression of *ZmCAAX* was significantly downregulated (Figure S6b). To assess the biological role of *ZmCAAX* in drought resistance, we compared drought stress responses between wild‐type and *Zmcaax*‐mutant plants. Seven days after germination, plants were subjected to 300 mM mannitol and drought‐related phenotypes were evaluated 2 weeks later. The *Zmcaax* mutants exhibited significantly higher shoot and root biomass than wild‐type plants (Figure 5a–c). Analysis of belowground traits following drought treatment revealed that maximum root length, root number, root weight, root volume and plant height were all significantly greater in *Zmcaax* mutants compared to wild‐type plants (Figure 5d; Figure S6c–g). After drought stress, the survival rates of *Zmcaax‐1* and *Zmcaax‐2* mutants were significantly higher (65.33% and 62.28%, respectively) than wild‐type plants (Figure 5e). Water loss analysis in detached leaves confirmed that *Zmcaax‐1* and *Zmcaax‐2* mutant plants retained water more efficiently than wild‐type plants (Figure 5f). Similarly, ion leakage was considerably lower in *Zmcaax* mutants than in wild‐type plants following 300 mM mannitol treatment (Figure 5g). Further analysis revealed that *Zmcaax* mutants had smaller stomatal apertures than wild‐type plants under both drought and normal conditions (Figure 5h–j). This finding suggests that *Zmcaax* mutants resist drought stress by reducing stomatal opening, which limits water loss.

Malondialdehyde (MDA) is a key marker of oxidative damage under drought stress, whereas antioxidant enzymes such as superoxide dismutase (SOD) and catalase (CAT) mitigate this damage by scavenging ROS (Zhou *et al*., 2022). Under drought stress, the activities of these antioxidant enzymes were significantly higher in *Zmcaax* mutants compared with wild‐type plants, while MDA content was significantly lower (Figure 5k–m). We also examined the expression levels of several genes encoding enzymes involved in ROS generation (*Zm00001d036557*, an NAD(P)H dehydrogenase) and ROS scavenging (*Zm00001d002436*, an alternative oxidase; *Zm00001d014608*, a peroxidase; and *Zm00001d036135*, a SOD). These genes exhibited differential expression in *Zmcaax* mutants relative to wild‐type plants, consistent with the increased ROS levels observed in *Zmcaax‐1* and *Zmcaax‐2* mutants (Figure 5n; Figure S6h–j). These findings suggest that *Zmcaax* mutants enhance drought tolerance by activating antioxidant defence mechanisms and reducing oxidative damage.

To further determine whether the function of the gene *CAAX* in regulating drought stress is conserved across plant species, we identified two homologous proteins, *NTAB0938690* and *NTAB0984080*, in tobacco. Both proteins showed a high degree of conservation in the CAAX domain (Figure S7a). We silenced the gene *NTAB0984080* through RNA interference (RNAi) and generated two transgenic lines, *NtCAAX‐RNAi‐1* and *NtCAAX‐RNAi‐2*. qRT‐PCR analysis confirmed that the expression levels of *NtCAAX* in the T_0_ generation were reduced by approximately 39%–45% compared with the wild‐type plants (Figure S7b). Then, the drought stress experiments were conducted on wild‐type and *NtCAAX‐RNAi* plants. After 15 days of water deficit, wild‐type plants exhibited wilting symptoms, whereas the two RNAi‐silenced lines, *NtCAAX‐RNAi‐1* and *NtCAAX‐RNAi‐2*, continued to grow normally without visible signs of wilting. Following water deficit and subsequent rehydration, the wild‐type plants completely died due to drought stress, whereas the *NtCAAX‐RNAi* plants survived (Figure S7c–h). These results indicate that silencing *CAAX* homologues enhances drought tolerance, suggesting that the negative regulatory role of *CAAX* in drought response is conserved between maize and tobacco.

### *ZmCAAX* modulates drought‐responsive gene expression under drought stress

**Figure 6:**
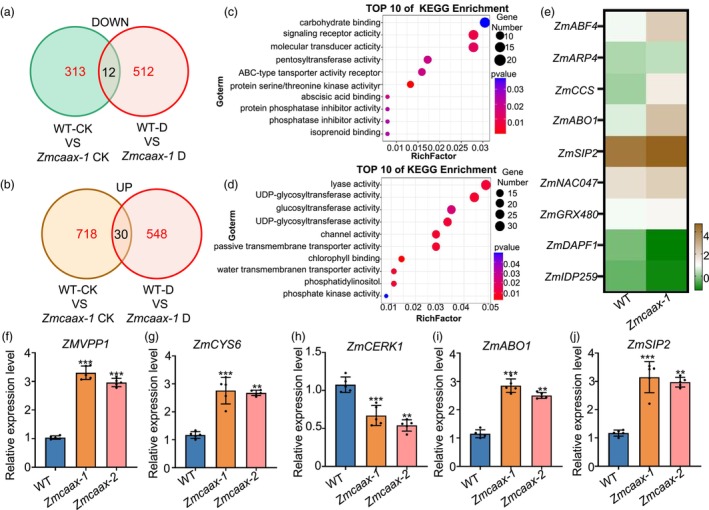
*ZmCAAX* regulates the expression of drought stress response genes. (a, b) Venn diagram illustrating differentially expressed genes (DEGs) in wild‐type and *Zmcaax‐1* mutant plants before and after drought treatment. Common DEGs between the two groups under control and drought conditions are highlighted. ‘D’ indicates drought stress. (c, d) KEGG analysis of downregulated genes (c) and upregulated genes (d) in *Zmcaax‐1* mutants compared with wild‐type plants after drought treatment. (e) Heatmap comparing expression levels of drought stress‐responsive genes in wild‐type controls and *Zmcaax‐1* mutants. (f–j) qRT‐PCR validation of drought stress‐related genes *ZmVPP1* (f), *ZmCYS6* (g), *ZmCERK1* (h), *ZmABO1* (i) and *ZmSIP2* (j) in WT and *Zmcaax‐1* mutants. Data are means ± SD of three biological replicates. Asterisks indicate significant differences, *P < 0.05, **P < 0.01, ***P < 0.001, as determined by a two‐sided Student's *t*‐test.

To elucidate the molecular mechanism of *ZmCAAX* under drought stress, we conducted RNA‐seq analysis on 24‐day‐old wild‐type and *Zmcaax*‐mutant plants subjected to drought stress. Samples were collected from three independent experiments under both control and drought conditions. A total of 1102 DEGs were identified, including 578 downregulated and 524 upregulated genes. KEGG analysis revealed that these DEGs were primarily associated with stress responses, carbohydrate binding, signalling receptor activity and molecular transducer activity (Table S2; Figure 6a–d). Further analysis indicated that the expression of most drought‐inducible genes was upregulated in *Zmcaax* mutants compared to wild‐type (Figure 6e–j). These results support the role of *ZmCAAX* as a negative regulator of drought resistance in maize.

### Overexpression of *ZmCAAX* confers drought sensitivity in maize

**Figure 7:**
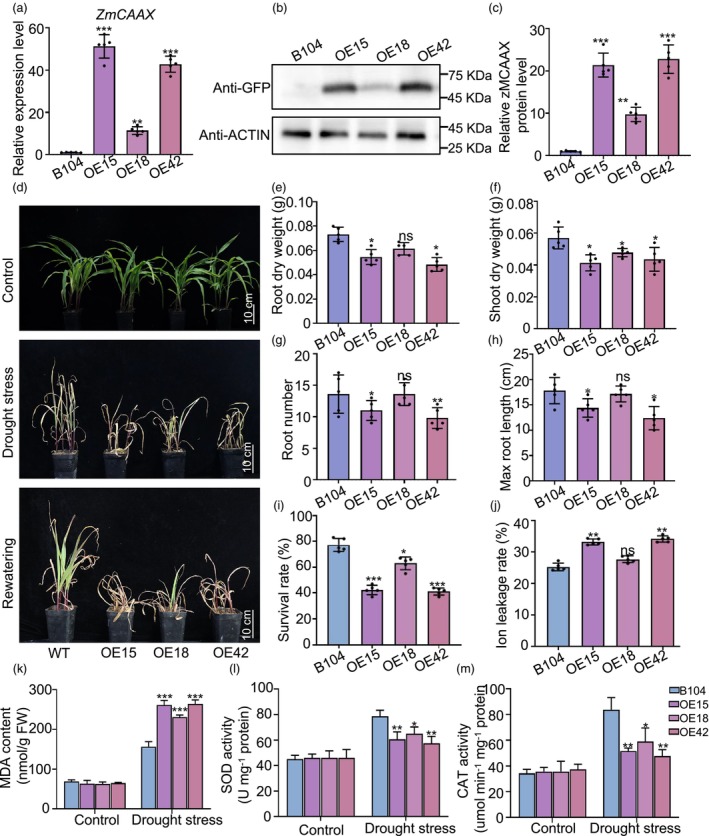
*ZmCAAX*‐overexpressing plants are sensitive to drought stress. (a) qRT‐PCR analysis of *ZmCAAX* expression levels in transgenic lines. Data are means ± SD of three biological replicates. Asterisks indicate significant differences: **P < 0.01, ***P < 0.001, as determined by a two‐sided Student's *t*‐test. (b) Immunoblotting analysis of ZmCAAX protein levels in transgenic lines. Total protein was extracted from leaves and ACTIN was used as a loading control. (c) Normalized plot of the ZmCAAX protein expression levels shown in (b) based on band intensities. Data are means ± SD of three biological replicates. (d) Representative images of wild‐type and *ZmCAAX‐*overexpressing plants grown in pots for 10 days post‐germination. Plants were subjected to drought treatment for 2 weeks and rehydrated for 3 days. Scale bar = 10 cm. (e–h) Quantitative description of phenotypes, including root dry weight (e), shoot dry weight (f), root number (g) and max root length (h), under drought stress. Data are means ± SD of three biological replicates. Asterisks indicate significant differences: *P < 0.05, as determined by a two‐sided Student's *t*‐test. (i, j) Survival rate (i) and ion leakage (j) of wild‐type and *ZmCAAX*‐overexpressing plants after drought stress. Data are means ± SD of three biological replicates. Asterisks indicate significant differences: *P < 0.05, **P < 0.01, as determined by a two‐sided Student's *t*‐test. (k–m) Determination of the MDA contents (k) and SOD activities (l) and CAT activities (m) in wild‐type and *ZmCAAX*‐overexpressing plants before and after drought treatment. Data are means ± SD of three biological replicates. Asterisks indicate significant differences: *P < 0.05, **P < 0.01, ***P < 0.001, as determined by a two‐sided Student's *t*‐test.

To further investigate the function of *ZmCAAX*, we generated transgenic maize plants that expressed *ZmCAAX* cDNA under the control of the *ZmUbi* promoter. Three independent *ZmCAAX*‐overexpressing lines (*ZmCAAX‐OE15*, *ZmCAAX‐OE18* and *ZmCAAX‐OE42*) were produced, which exhibited significantly higher *ZmCAAX* transcript and protein levels than wild‐type plants (Figure 7a–c). We then assessed the drought response of these transgenic plants. Compared with wild‐type plants, *ZmCAAX‐OE* plants were more sensitive to drought and drought treatment resulted in reduced root length, shoot and root biomass and survival rate (Figure 7d–i). Under drought stress, *ZmCAAX‐OE* plants exhibited significantly higher ion leakage than wild‐type plants (Figure 7j). Similarly, under drought stress, MDA content increased in wild‐type plants and was further exacerbated in *ZmCAAX‐OE* plants, accompanied by significantly greater reductions in SOD and CAT activities in the transgenic lines compared with wild‐type plants. In contrast, no significant differences in enzyme activities were observed between wild‐type and transgenic plants under control conditions (Figure 7k–m). These results demonstrate that *ZmCAAX* overexpression exacerbates oxidative damage induced by drought stress in maize. Collectively, our findings suggest that *ZmCAAX* primarily regulates cellular detoxification and reactive oxygen species (ROS) scavenging to enhance drought adaptation in maize.

### ZmCAAX interacts with the ABA biosynthetic enzyme ZmNCED3

**Figure 8:**
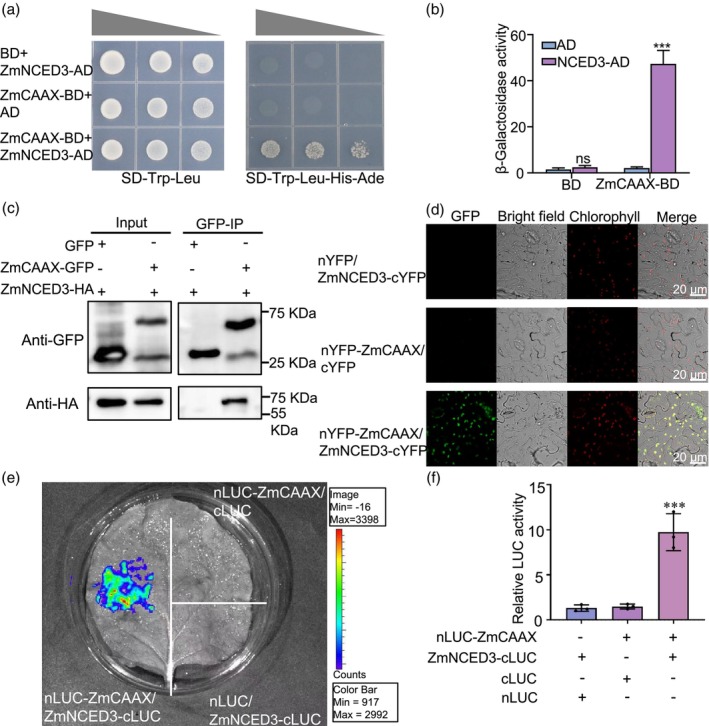
ZmCAAX interacts with ZmNCED3. (a) Yeast two‐hybrid (Y2H) assay showing the interaction between ZmCAAX and ZmNCED3. Yeast cells harbouring the indicated plasmid combinations were grown on nonselective (SD/‐Trp/‐Leu) and selective (SD/‐Trp/‐Leu/‐His/‐Ade) media. Cells were diluted in three concentrations from left to right and photos were taken after 5 days. (b) Quantification of β‐galactosidase (β‐Gal) activity in yeast cells from (a). Data are presented as the means ± SD of three biological replicates. Asterisks indicate significant differences: ***P < 0.001, as determined using two‐sided Student's *t*‐test. (c) Coimmunoprecipitation (Co‐IP) assay showing the *in vivo* interaction between ZmCAAX and ZmNCED3. Construct combinations were expressed in *Nicotiana benthamiana* leaves. Total proteins were extracted and immunoprecipitated with anti‐GFP agarose beads. The proteins were detected using anti‐GFP and anti‐NCED3 antibodies. (d) Bimolecular fluorescence complementation (BiFC) assay demonstrating the interaction between ZmCAAX and ZmNCED3. Yellow fluorescence indicates positive interactions. cYFP and nYFP were used as negative controls. Scale bar = 20 μm. (e) Luciferase complementation imaging (LCI) assay analysing the interaction between ZmNCED3 and ZmCAAX in *N. benthamiana*. The colour bar below indicates the range of luminescence intensity in each image. (f) Quantification of LUC activity in *N. benthamiana* leaves from (e). Minus symbols (−) indicate empty vectors for nLUC and cLUC. Data are means ± SD of three biological replicates. Asterisks indicate significant differences: ***P < 0.001, as determined by a two‐sided Student's *t*‐test.

To investigate the molecular mechanism by which ZmCAAX regulates plant drought responses, we performed a yeast two‐hybrid (Y2H) assay and identified ZmNCED3 as an interacting partner of ZmCAAX (Figure 8a). The interaction between ZmCAAX and ZmNCED3 was confirmed by measuring β‐galactosidase (β‐gal) activity (Figure 8b). To further validate this interaction, we conducted coimmunoprecipitation (Co‐IP) assays by coexpressing ZmCAAX‐GFP with ZmNCED3‐HA in *Nicotiana benthamiana* leaves. These results confirmed the physical interaction between ZmCAAX and ZmNCED3 (Figure 8c). Furthermore, bimolecular fluorescence complementation (BiFC) assays revealed that the interaction between ZmCAAX and ZmNCED3 primarily occurred in the chloroplasts (Figure 8d). To further validate the interaction, luciferase complementation imaging (LCI) assays were performed in *N. benthamiana*. Coexpression of ZmNCED3‐cLUC and ZmCAAX‐nLUC resulted in strong luciferase activity, with a significant increase compared to controls (Figure 8e,f). Taken together, these results demonstrate that ZmCAAX interacts with ZmNCED3 both *in vitro* and *in vivo*.

Cis‐epoxycarotenoid dioxygenase (NCED) is a key rate‐limiting enzyme in ABA biosynthesis (Chen *et al*., 2023c; Li *et al*., 2024). In maize, 14 members of the *ZmNCED* gene family have been identified (Figure S8a). To assess the specificity of the ZmCAAX‐ZmNCED3 interaction, we conducted an additional Y2H assay. The results confirmed that ZmCAAX did not interact with ZmVP14, the most homologous member of the ZmNCED family, indicating that the interaction between ZmCAAX and ZmNCED3 is highly specific (Figure S8).

### ZmCAAX promotes the degradation of ZmNCED3 and regulates ABA synthesis

**Figure 9:**
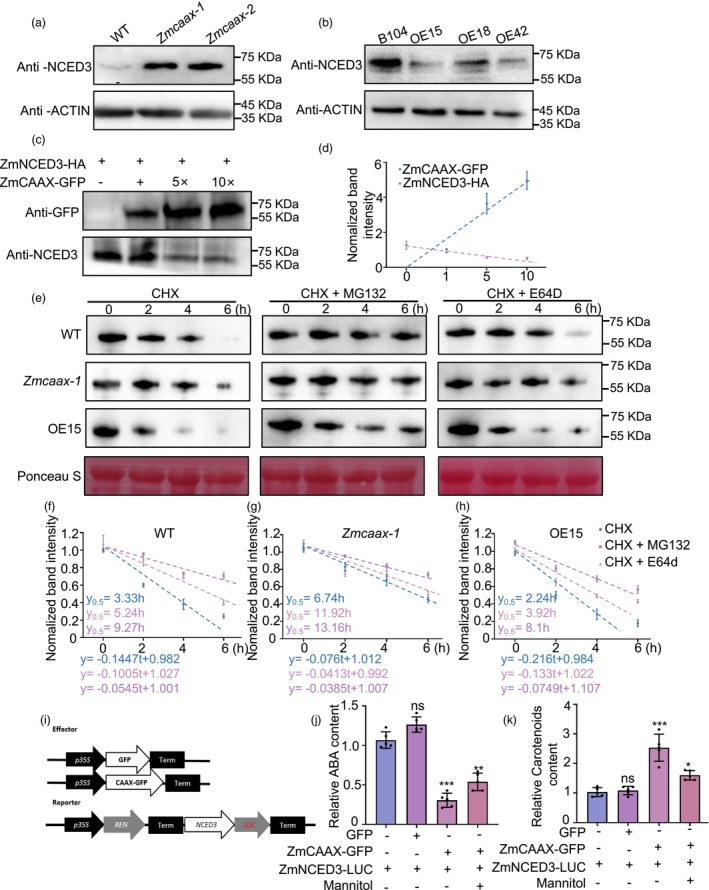
ZmCAAX regulation of ZmNCED3 protein stability. (a, b) Immunoblotting analysis of ZmNCED3 protein levels in wild‐type, *Zmcaax* mutants and *ZmCAAX*‐overexpressing plants. Total protein was extracted from phenotypic leaves, with ACTIN serving as a loading control. (c) Immunoblot analysis of ZmNCED3‐HA levels in *Nicotiana benthamiana* leaves coinfiltrated with ZmCAAX‐GFP at different concentrations. Constructs were expressed transiently and proteins were detected using anti‐GFP and anti‐NCED3 antibodies. (d) Normalized plot of ZmCAAX and ZmNCED3 contents based on the band intensities shown in (c). Error bars indicate SEM (*n* = 3). (e) Immunoblot analysis comparison of ZmNCED3 degradation between the WT, *Zmcaax, ZmCAAX‐OE15*. Two‐week‐old seedlings were treated with 100 μM cycloheximide (CHX), 100 μM CHX + 100 μM MG132 and 100 μM CHX + 100 μM E64d for different times. Total proteins were extracted and used for immunoblotting analysis with anti‐NCED3 antibody. (f–h) Normalized plot of ZmNCED3 contents based on the band intensities shown in (e). Error bars indicate SEM (*n* = 3). (i) Schematic diagram of constructs used in dual‐luciferase transient activity assays to assess the regulatory effects of ZmCAAX on ZmNCED3 expression. (j, k) Cotransfection of ZmCAAX‐GFP and ZmNCED3‐HA proteins in *N. benthamiana* with and without 300 mM mannitol (Man). Carotenoid and ABA contents in the leaves were determined. Data are means ± SD of three biological replicates. Asterisks indicate significant differences: *P < 0.05, **P < 0.01, ***P < 0.001; ns indicates no significant difference *P* ≥ 0.05.

ZmNCED3 is a key regulator of the drought stress response (Sato *et al*., 2018). To investigate the genetic relationship between *ZmCAAX* and *ZmNCED3*, we analysed ZmNCED3 expression at both the transcript and protein levels. No significant differences in *ZmNCED3* transcript levels were detected among wild‐type, *Zmcaax* mutants and *ZmCAAX‐OE* lines (Figure S9a,b). However, ZmNCED3 protein levels were significantly elevated in *Zmcaax* mutants but reduced in *ZmCAAX‐OE* lines. Notably, the decrease in ZmNCED3 protein levels in overexpression lines was negatively correlated with ZmCAAX expression levels (Figure 9a,b). We also compared the enzymatic activity of ZmNCED among wild‐type, *Zmcaax* mutants and *ZmCAAX‐OE* plants. The results revealed no significant differences in the enzymatic activity of ZmNCED among these groups (Figure S9c,d). As ZmCAAX accumulated in *N. benthamiana*, ZmNCED3 protein levels progressively declined (Figure 9c,d). These findings suggest that ZmCAAX regulates ZmNCED3 degradation, potentially influencing its stability.

To verify this hypothesis, we conducted cell‐free degradation assays on wild‐type, *Zmcaax‐1* and *ZmCAAX‐OE15* plants by treating them with the protein synthesis inhibitor cycloheximide (CHX), the proteasome inhibitor MG132 (which inhibits the 26S proteasome degradation pathway) and the cysteine protease inhibitor E64d (which inhibits the vacuolar degradation pathway) to assess their effects on ZmNCED3 degradation (Figure 9e). The half‐life of ZmNCED3 was 3.33 h in wild‐type plants but decreased to 2.24 h in *ZmCAAX‐OE15*, indicating significantly accelerated degradation of ZmNCED3. Although E64d partially inhibited ZmNCED3 degradation, MG132 exhibited a stronger inhibitory effect (Figure 9f–h). These data directly demonstrate that ZmCAAX‐mediated ZmNCED3 degradation is primarily dependent on the 26S proteasome pathway, with the cysteine protease pathway also contributing to this process.

To assess whether the interaction between ZmCAAX and ZmNCED3 is essential for ABA biosynthesis, we performed a dual‐luciferase transient expression assay in maize leaf protoplasts. Using the 35S promoter, we coexpressed *ZmNCED3* with the REN internal control and the LUC reporter gene. When ZmCAAX was coexpressed, ABA levels significantly decreased, while carotenoid levels increased relative to the control group. However, these differences decreased with the application of mannitol (Figure 9i–k). Additionally, we measured the ABA levels in wild‐type, *Zmcaax* mutants and *ZmCAAX‐OE* plants. *Zmcaax* mutants accumulated significantly higher ABA levels than wild‐type plants. Drought stress induced ABA biosynthesis, leading to significantly higher ABA accumulation in *Zmcaax* mutants compared with wild‐type plants. In contrast, ABA levels were lower in *ZmCAAX‐OE* plants than in wild‐type plants (Figure S9e,f). Notably, *ZmCAAX* overexpression led to increased carotenoid accumulation (Figure S10). These findings suggest that ZmCAAX modulates carotenoid degradation and ABA biosynthesis by regulating ZmNCED3 protein stability.

## Discussion

### ZmCAAX regulates chloroplast protein ZmNCED3 degradation under drought stress

Chloroplast‐associated protein degradation (CHLORAD) is essential for plant development and response to abiotic stress (Eckardt *et al*., 2024; Ling *et al*., 2019). In *Arabidopsis*, deficiency of the RING E3 ubiquitin ligase suppressor of ppi1 locus 1 (SP1) results in leaf yellowing (Ling *et al*., 2019, 2021). In tomato, the homologous protein SPL1 promotes the chloroplast‐to‐chromoplast transition and regulates fruit ripening (Ling *et al*., 2021). Chloroplast protein degradation is regulated by multiple pathways, including the ubiquitin‐proteasome system (UPS), the 26S proteasome and autophagy (Guo *et al*., 2021; Li and Jarvis, 2024; Sun *et al*., 2023). The cysteine protease PAP14 contributes to chloroplast protein degradation, thereby regulating leaf senescence (Frank *et al*., 2019). In tomato, the E3 ubiquitin ligase COP9 signalosome subunit 5A (SICSN5A) is essential for maintaining chloroplast protein homeostasis (Lu *et al*., 2024). However, the regulatory mechanisms controlling CHLORAD under developmental and environmental cues remain largely unknown (Eckardt *et al*., 2024).

**Figure 10:**
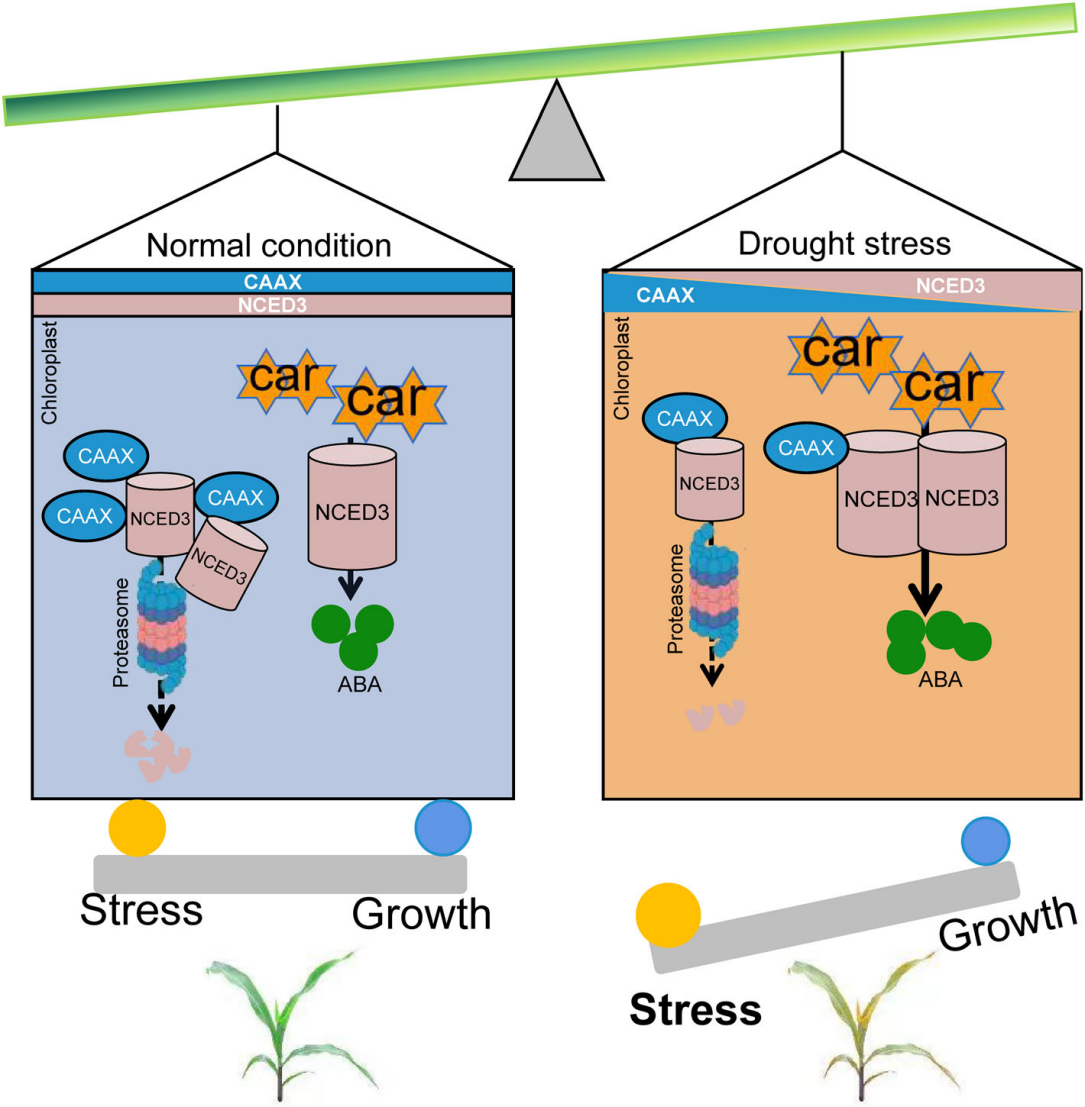
Model of ZmCAAX regulation of ABA content and maize drought stress response. Under normal conditions, ZmCAAX interacts with ZmNCED3, promoting its degradation and thereby maintaining stable carotenoid and abscisic acid (ABA) levels, which regulate plant growth and development. In contrast, under drought stress, ZmCAAX expression is downregulated, resulting in reduced degradation of ZmNCED3. Consequently, ZmNCED3 accumulates, promoting the conversion of carotenoids into ABA. The resulting ABA accumulation induces stomatal closure, ultimately enhancing maize drought resistance.

The CAAX motif serves as a key signal for post‐translational modifications, regulating the localization and stability of various proteins (Bracha‐Drori *et al*., 2008; Manolaridis *et al*., 2013; Zou *et al*., 2024). In mice, UBL3, a CAAX domain‐containing protein, mediates protein sorting into small extracellular vesicles through post‐translational modification (Ageta *et al*., 2018). Similarly, in animals, the E3 ubiquitin ligase FBXL2 utilizes its CAAX motif to facilitate EGFR degradation (Li *et al*., 2023; Niu *et al*., 2021). Although the involvement of CAAX family proteins in protein degradation is well established in other species, their role in regulating protein stability in plants remains largely unknown. In *Arabidopsis*, the CAAX motif protein BCM1 modulates chloroplast protein SGR1 degradation, delaying chlorophyll breakdown (Wang *et al*., 2020a). Notably, in maize, ZmCAAX regulates the degradation of the chloroplast‐localized ABA biosynthetic enzyme ZmNCED3, thereby modulating carotenoid and ABA levels and ultimately affecting plant growth, development and drought resistance (Figure 10). These findings suggest that CAAX proteins play a conserved role in regulating chloroplast‐associated protein degradation across plant species. However, the precise molecular mechanism by which ZmCAAX regulates ZmNCED3 degradation remains to be elucidated. Specifically, it remains unclear whether ZmCAAX functions as an E3 ubiquitin ligase that directly mediates protein degradation, recruits an E3 ligase to facilitate ZmNCED3 degradation or influences its prenylation to regulate stability. Further studies are needed to elucidate these mechanisms at the molecular level.

### *ZmCAAX* links leaf yellowing and drought resistance

Under drought stress, maize leaves exhibit reduced photosynthesis and growth arrest, along with increased respiration and transport of photosynthetic products, which is accompanied by decreased chlorophyll content and leaf yellowing (Dong *et al*., 2020; Gupta *et al*., 2020; Waadt *et al*., 2022). These physiological changes are believed to mitigate water loss by limiting transpiration and improving water use efficiency (Gupta *et al*., 2020; Wang *et al*., 2024a). In *Arabidopsis*, mutations in the E3 ubiquitin ligase NOT4A disrupt chloroplast development, leading to leaf yellowing and dwarfism. Interestingly, these mutants exhibit enhanced stress resistance, suggesting a trade‐off between growth and stress adaptation (Bailey *et al*., 2021, 2022). Similarly, mutations in CSN5A result in dwarfism and leaf yellowing but confer strong resistance to salt stress in *Arabidopsis* (Singh *et al*., 2019, 2021; Zhou *et al*., 2021). Here, we identify *ZmCAAX* as a key regulator of leaf pigmentation and drought stress responses in maize. We provide the first evidence that *Zmcaax* leaf yellowing mutants exhibit enhanced adaptation to drought stress.

Does a direct link exist between leaf yellowing and plant drought resistance? Several studies suggest that chloroplasts and chromoplasts play a significant role in regulating leaf colour (Ling *et al*., 2021). Under drought stress, decreased chlorophyll content leads to reduced photosynthesis, potentially mitigating self‐inflicted oxidative damage (Gupta *et al*., 2020; Razi and Muneer, 2021). Excess energy generated during photosynthesis leads to ROS accumulation, which damages plant cells and exacerbates drought‐induced stress (Teardo *et al*., 2019; Wang *et al*., 2020a). During drought stress and the maturation phase, an increasing number of chloroplasts are converted into chromoplasts and other plastids. These plastids, which are highly conserved throughout evolution, are increasingly recognized for their role in retrograde signalling to the nucleus under abiotic stress conditions (Ling *et al*., 2021; Llorente *et al*., 2020). SEM analysis revealed degradation of chloroplasts and accumulation of plastids in the *Zmcaax* mutant. However, whether *ZmCAAX* directly regulates this transformation remains unclear, shedding light on the broader regulatory networks of chloroplast functionality under stress.

### *ZmCAAX* and *ZmNCED3* have undergone differential selection during domestication

Recent studies indicate that long‐term drought stress can reduce plant chlorophyll content, a process influenced by selection pressures during domestication (Chen *et al*., 2023b; Gupta *et al*., 2020; Han *et al*., 2024). Domestication, regarded as the earliest form of crop breeding, is estimated to have shaped the genetic diversity of most crops (Acosta‐Bayona *et al*., 2024; Yang *et al*., 2023). Under drought stress, genetic traits that confer adaptability to harsh conditions often originate from the natural variation present in wild relatives, providing a valuable resource for crop improvement (Bartlett *et al*., 2023). During prolonged drought periods, numerous genes undergo selection, with beneficial alleles typically retained while deleterious alleles are purged (Berube *et al*., 2024). It has been reported that maize retains, on average, only 57.1% of the nucleotide diversity observed in teosinte (Wright *et al*., 2005). To investigate whether *ZmCAAX* underwent selection during maize domestication, we calculated the nucleotide diversity of *ZmCAAX* in improved maize lines, landraces and teosinte by using HapMap 3 data (Bukowski *et al*., 2018). The analysis showed high nucleotide diversity in improved maize lines but a significant reduction in teosinte, suggesting that *ZmCAAX* underwent negative selection during maize domestication (Figure S11a). Additionally, we performed a selection analysis on *ZmNCED3*, which revealed evidence of positive selection (Figure S11b). This suggests that the drought resistance gene *ZmNCED3* was selected against during maize domestication and breeding.

To assess the relationship between natural variations in *ZmCAAX* and *ZmNCED3* with drought resistance, we performed a candidate gene association study using a maize panel of 220 inbred lines. Chlorophyll content was measured before and after drought stress treatment. The analysis revealed a significant correlation between *ZmCAAX* and *ZmNCED3* expression levels and their responses to drought stress. Selective domestication analysis indicated that *ZmCAAX* was subject to purifying selection, whereas *ZmNCED3* underwent positive selection, likely in response to abiotic stresses such as high temperature and drought. These evolutionary pressures may have enhanced maize adaptation to environmental challenges (Figure S11c,d). The contrasting selection patterns of *ZmCAAX* and *ZmNCED3* highlight their distinct roles in drought stress responses and underscore their importance in maize domestication and environmental adaptation. The observed differential selection patterns negative for *ZmCAAX* and positive for *ZmNCED3* underscore their significance in maize domestication and adaptation to environmental challenges.

### *ZmCAAX* modulates the trade‐off between drought resistance and grain yield

Numerous studies have established that ABA is a key regulator of abiotic stress resistance (Qiu *et al*., 2021; Waadt *et al*., 2022; Xie *et al*., 2024). ABA accumulation in response to drought stress not only enhances drought resistance but also plays a crucial role in grain development (Ma *et al*., 2023; Yang *et al*., 2022). Recent studies suggest that ABA signalling regulates grain development and filling, where elevated ABA levels promote grain filling, increase grain weight and influence grain morphology (Chen *et al*., 2024b; Ma *et al*., 2023; Wang *et al*., 2024b). Under drought stress, ABA levels likely mediate the trade‐off between drought resistance and grain yield (Wang *et al*., 2024b). In *Zmcaax* mutants, elevated ABA levels, reduced stomatal density, decreased stomatal aperture and lower transpiration rates collectively enhanced drought resistance. However, impaired chloroplast development limited photosynthetic efficiency, ultimately leading to reduced grain yield. Fine‐tuning *ZmCAAX* expression represents a promising strategy for balancing drought resistance and carotenoid accumulation in maize, with potential applications for improving grain quality.

Based on our findings, we propose that crop design tailored to specific environmental conditions can contribute to a sustainable food supply from both ecological and economic perspectives. In agricultural regions with ample rainfall or irrigation, upregulating *ZmCAAX* expression may enhance carotenoid accumulation and improve yield. Conversely, in water‐limited environments, downregulating *ZmCAAX* expression may improve drought adaptation. Future studies should focus on identifying superior haplotypes and non‐synonymous variants of *ZmCAAX* that contribute to drought resistance. Selecting suitable genotypes could help maintain drought tolerance while mitigating unfavourable traits such as reduced photosynthetic efficiency or yield penalties. Additionally, identifying upstream regulators of *ZmCAAX* and its promoter, along with employing CRISPR/Cas9, RNAi and EMS mutagenesis, could facilitate the fine‐tuning of its expression. Moreover, integrating gene editing technologies and incorporating a drought‐repressed promoter could ensure that *ZmCAAX* maintains normal expression under favourable conditions while being repressed under drought stress. The above strategy can optimize the balance between yield stability and stress resistance, paving the way for improving the adaptability of maize in different agricultural landscapes.

## Materials and methods

### Plant materials and growth condition

Approximately 2 mL of wild‐type (RP125) pollen was treated with a 0.066% EMS solution (Sigma‐Aldrich, M0880, Saint Louis, MO) prepared in mineral oil (Sigma‐Aldrich, M8410, Saint Louis, MO). The mixture was vigorously shaken to suspend the pollen and subsequently incubated for 45 min, with continuous or frequent shaking every minute to ensure uniform pollen suspension. After incubation, the treated pollen was used to pollinate the ears of RP125 following the protocol described by (Nie *et al*. (2021)). An F_2_ segregating population was generated by crossing *Zmcaax‐1* mutants (M2 generation) in the RP125 background with B73. The *Zmcaax‐2* allele was identified from our EMS‐induced mutant library (http://emsdb.sdau.edu.cn). Both the *Zmcaax‐1* and *Zmcaax‐2* alleles were backcrossed into the RP125 inbred line for over five generations to ensure genetic stability.

Three independent *ZmCAAX* overexpression lines (*ZmUbi::ZmCAAX‐OE*) were developed in the B104 inbred background. Nontransgenic and overexpression maize lines were cultivated in experimental fields in Taian, Shandong, China (35.6°N, 117.5°E) and Sanya, Hainan, China (18.2°N, 109.3°E). To examine morphogenesis and drought stress phenotypes at the seedling stage, *Zmcaax* mutants and *ZmCAAX‐OE* lines were grown in a controlled growth chamber (Greenfuture Envirotech, Shanghai, China) under a 14‐h light/10‐h dark cycle at 25 °C, with light intensity maintained at approximately 400 μmol/m^2^/s.

### Drought resistance phenotype analysis

All physiological experiments were performed following previously published protocols with minor modifications (Gao *et al*., 2022; Xiang *et al*., 2024). To assess drought‐stress phenotypes, *Zmcaax* mutant and *ZmCAAX‐OE* seedlings were treated with 300 mM mannitol (Solarbio, Beijing, China) at 12 days after germination. After approximately 10 days of treatment, survival rates were recorded and leaf electrolyte leakage was measured. For root trait analysis, seedlings were cultured in aqueous solutions containing 300 mM mannitol or control conditions without mannitol. After 10 days, root volume and surface area were analysed using WinRhizo Pro 2008a (Regent Instruments Inc., Quebec, Canada) in conjunction with a professional scanner (Epson XL 10000; Epson, Japan).

### Generation of *ZmCAAX* transgenic plants

To generate *ZmCAAX‐OE* plants, the *ZmCAAX* coding sequence (CDS) was amplified from a cDNA library of B73 (AGPv4) using primers listed in Table S3. The amplified sequence was cloned into the pCAMBIA3301‐GFP vector under the control of the maize *ZmUbi* promoter. The plasmid containing the *ZmCAAX* insert was transformed into *Agrobacterium tumefaciens* strain HA105. All these recombinant vectors were *Agrobacterium*‐transformed into maize inbred line B104. T_0_, T_1_ and T_2_ plants were grown in a greenhouse under a 14‐h light/10‐h dark cycle. Transgenic‐positive and wild‐type sibling plants were identified in each generation through *ZmCAAX‐OE*‐specific PCR analysis, using primers provided in Table S3.

To generate RNAi lines of *NtCAAX*, the HD inbred background was utilized. Forward and reverse RNAi fragments were amplified with gene‐specific primers. The target fragment was ligated into the *pHANNIBAL* vector to construct *Ntcaax*‐*pHANNIBAL*. The construct was subsequently ligated into the destination NotI‐digested vector *pART27*, generating the *NtCAAX‐RNAi* vector. Detailed methods for vector construction can be found in (Wesley *et al*., 2001).

### Map‐based cloning of *ZmCAAX*

The F_2_ segregating population was generated by crossing *yp1* mutants in the RP125 background with B73. Preliminary screening of linkage markers was performed using the F_2_ population and a BSA strategy (Gallavotti and Whipple, 2015). DNA samples were pooled from 40 *yp1* mutants and 40 wild‐type plants from the F_2_ population. For map‐based cloning, DNA from individual F_2_ mutant plants was used for fine mapping. Fine‐mapping markers were designed based on sequence polymorphisms between the RP125 and B73 genomes. To identify the mutated gene, the gene was amplified from both wild‐type and mutant plants and subjected to Sanger sequencing. Primer sequences are listed in Table S3.

### Subcellular localization

To determine the subcellular localization of *ZmCAAX*, the CDS of *ZmCAAX* without the stop codon was inserted into the *pROKII* vector at the *Bam*HI and *Kpn*I restriction sites, positioned between the 35S cauliflower mosaic virus (*CaMV*) promoter and the GFP. The resulting fusion construct was transformed into *A. tumefaciens* strain *GV3101*. Both the ZmCAAX‐GFP fusion vector and the GFP control vector were transiently expressed in maize mesophyll protoplasts. After 12 h of incubation in darkness, the subcellular localization of ZmCAAX‐GFP was analysed using GFP and the fluorescence signals were detected using a confocal laser‐scanning microscope (LSM880; Zeiss, Oberkochen, Germany).

### RNA extraction and qRT‐PCR

Total RNA was extracted using the TRIzol reagent (Tiangen, Beijing, China) and purified with the RNAsimple Mini Kit (Tiangen, Beijing, China). After DNaseI digestion, cDNA synthesis was performed using the PrimeScript RT Reagent Kit with gDNA Eraser kit (Accurate Biology, Hunan, China). For qRT‐PCR, approximately 80–100 ng of cDNA from each sample was used as a template. Reactions were carried out using SYBR Green (Accurate Biology Hunan, China) on a Bio‐Rad CFX96 Real‐Time PCR System (Bio‐Rad Laboratories, Hercules).

### Physiological experiments

All the physiological experiments were performed as described previously with some modifications (Gao *et al*., 2022; Xiang *et al*., 2024). The water loss rate of detached leaves from wild‐type, *Zmcaax* mutants and *ZmCAAX*‐OE transgenic plants was determined by measuring the fresh weight loss at different time points. Specifically, the first true leaf from 21‐day‐old seedlings was detached and immediately weighed. The detached leaves were then maintained at room temperature and reweighed at 1‐h intervals. The experiment was conducted with five biological replicates.

### Protein–protein interaction assays

Y2H, LCI, BiFC and Co‐IP assays were used to measure the interaction between ZmCAAX and ZmNCED3. Detailed procedures are provided in Text S1.

### Immunoblotting analysis and SDS‐PAGE

For immunoblotting and SDS‐PAGE analysis, total proteins were extracted from wild‐type, *Zmcaax* mutants and *ZmCAAX‐OE* seedlings using a protein extraction buffer (Solarbio, Beijing, China). Protein concentrations were quantified using a Bio‐Rad protein assay kit (Solarbio, Beijing, China). The samples were mixed with 5 × SDS loading buffer and boiled for 5 min. Proteins were separated on 10% SDS‐PAGE gels and transferred to nitrocellulose membranes following standard protocols (Yu *et al*., 2019). Anti‐NCED3 (Solarbio, Beijing, China; 1:1000 dilution) antibodies were used to detect ZmNCED3 expression in the samples. Anti‐ACTIN antibodies served as a loading control and anti‐GFP antibodies were used to detect ZmCAAX expression in *ZmCAAX‐OE* plants. For quantitative analysis, ZmNCED3 band intensity was normalized to ACTIN band intensity in each lane.

### Cell‐free assay and calculation of protein half‐life

To determine whether ZmCAAX regulates the degradation pathway of ZmNCED3, cell‐free degradation assays were conducted following previously described protocols with minor modifications (Yu *et al*., 2024; Zhou *et al*., 2021). Two‐week‐old seedlings were treated with 100 mM cycloheximide (CHX) to inhibit protein synthesis, followed by treatment with 100 mM MG132 or 100 mM E64d. Samples were collected every 2 h. Protein levels were detected using anti‐NCED3 antibodies. The intensity of each protein band was quantified using ImageJ software.

### Transient expression in *N. benthamiana*

The full‐length CDS of *ZmCAAX* was cloned into the *pBI121* overexpression vector to create the effector construct, while the full‐length CDS of *ZmNCED3* was inserted into the *pGreenII 0800‐LUC* vector, which contains the firefly luciferase (LUC) coding sequence, to generate the reporter construct. The *Renilla* luciferase (REN) gene, driven by the 35S CaMV promoter in the *pGreenII 0800‐LUC* vector, served as an internal control. These constructs were transiently expressed in *N. benthamiana* leaves through *Agrobacterium*‐mediated infiltration. The expression levels of *ZmNCED3* and the content of carotenoids and ABA were detected. Three biological replicates were obtained from four independent *N. benthamiana* plants. Primer sequences used in this study are provided in Table S3.

### Statistical analysis

Data were analysed and plotted using Origin 2021 (OriginLab), Excel (Microsoft 365) and GraphPad Prism v8.0.2. Statistical analyses for all experiments are described in the figure legends. Comparisons between two groups were performed using two‐tailed Student's *t*‐tests. For comparisons among three or more groups, one‐way analysis of variance (ANOVA) was conducted, followed by Tukey's honestly significant difference test. Statistical significance was determined based on *P* values, with the following notations: ns, no significant difference (*P* ≥ 0.05); *P < 0.05; **P < 0.01; ***P < 0.001. Panels of micrographs show representative results from one of three independent experiments.

## Conflict of interest

The authors declare no conflict of interest.

## Author contributions

XHL, BZ, JYD and SC conducted the majority of the molecular experiments; XHL, BZ, JYD, SC, YJW, QGL and SLZG developed genetic materials; XHL and SLZG performed RNA‐seq analysis; XZL, YXN, FX, AGY, ZMZ and HPD conceived the project and provided the material; XHL and ZMZ wrote the manuscript.

## Supporting information


**Figure S1** Phenotype characterization of the *yp1* mutants. (a) Wild‐type (RP125) and *yp1* mutants at four developmental stages, photographed at various time points post‐germination. (b) Quantification of pigment contents in the second leaf of wild‐type and *yp1* mutants, including Chlorophyll a (Chl a), chlorophyll b (Chl b), carotenoid (Car). Data are means ± SD of three biological replicates. Asterisks indicate significant differences: *P < 0.05, **P < 0.01, ***P < 0.001, ns indicates no significant difference *P* ≥ 0.05. (c, d) Comparative analysis of plant height (c) and flowering time (d) in wild‐type and *yp1* mutants plants. Data are means ± SD of three biological replicates. Asterisks indicate significant differences: **P < 0.01, ns indicates no significant difference *P* ≥ 0.05.
**Figure S2** Abnormal chloroplast development in the *yp1* mutants. (a) Quantification of epidermal structures, including macrohairs (mh), prickle hairs (ph), bicellular hairs (bh) and stomatal complexes (st), per 2.1 mm^2^ of the adaxial leaf surface in wild‐type and *yp1* mutants plants. Data are means ± SD (*n* = 10). Significant differences were determined by two‐way ANOVA. Asterisks indicate significant differences: *P < 0.05, **P < 0.01, ***P < 0.001. (b) Immunoblot analysis of key photosynthetic proteins in wild‐type and *yp1* mutants plants. RbcL (Rubisco large subunit), AtpB (ATP synthase β‐subunit) and PsbD (PSII reaction centre protein D2) were detected using specific antibodies. Serial dilutions (1, 0.5, 0.1) of total protein extracts were loaded for semi‐quantitative comparison. Ponceau S staining was used as a loading control. (c) qRT‐PCR analysis of photosynthetic complex subunit expression levels in wild‐type and *yp1* mutants plants. Data are presented as means ± SD of three biological replicates. Asterisks denote statistically significant differences: *P < 0.05, **P < 0.01, ***P < 0.001, as determined by Student's two‐tailed paired *t* tests.
**Figure S3** Genetic and phenotypic characterization of *ZmCAAX* in maize. (a, b) Phenotypic characterization of leaves at different positions: lower leaves (below the ear), ear leaves (at the ear position) and upper leaves (above the ear). Scale bar = 10 cm. (b) Chlorophyll a and chlorophyll b contents were quantified in leaves from the B73 × *yp1* F_2_ segregating population. Data are means ± SD of three biological replicates. Asterisks indicate significant differences: **P < 0.01, ***P < 0.001, as determined by Student's two‐tailed paired *t* tests. (c, d) Bulked segregant analysis (BSA) sequencing profiles showing the distribution of genetic variations associated with the *yp1* mutant phenotype. (e–g) *ZmCAAX* allele identification and sequencing results analysis. (h–j) Quantification of chlorophyll a (h), chlorophyll b (i) and total carotenoid (j) contents in wild‐type, *Zmcaax‐1*, *Zmcaax‐2* and *Zmcaax‐1* × *Zmcaax‐2* mutants. Data are means ± SD of three biological replicates. Asterisks indicate significant differences: **P < 0.01, as determined by Student's two‐tailed paired *t* tests.
**Figure S4** *Zmcaax* mutation inhibits seed germination. (a) Comparison of seed germination between wild‐type and *Zmcaax‐1* mutant seeds. Scale bar = 1 cm. (b) Germination rate over time in wild‐type and *Zmcaax‐1* mutant seeds. Data represent means ± SEM, Asterisks indicate significant differences: **P < 0.01, ***P < 0.001, as determined by Student's *t*‐test. (c, d) Oxygen consumption dynamics during germination in wild‐type and *Zmcaax*‐mutant seeds.
**Figure S5** Transcriptomic analysis and ABA quantification in *Zmcaax* mutants. (a) Principal component analysis (PCA) of transcriptomic data showing clustering of biological replicates for wild‐type and *Zmcaax*‐mutant plants. (b, c) GO functional enrichment analysis of genes involved in pathways related to molecular functions and cellular localization. Numbers represent the number of enriched genes. (d) Heatmap of MVA pathway gene expression in wild‐type and *Zmcaax*‐mutant plants, based on RNA‐seq data. (e) Determination of ABA content in wild‐type and *Zmcaax*‐mutant plants. Data are means ± SD of three biological replicates. Asterisks indicate significant differences: **P < 0.01, ***P < 0.001.
**Figure S6** *ZmCAAX* genes participate in maize drought tolerance. (a, b) qRT‐PCR analysis of *ZmCAAX* expression levels in response to 5 μM ABA (a) and 300 mM mannitol (b) treatment. Data are means ± SD of three biological replicates. (c–g) Morphological analysis of *Zmcaax‐1* and *Zmcaax‐2* mutants seedlings under drought stress. Root number (c), root fresh weight (d), root dry weight (e), root volume (f) and seedling height (g) were quantified in wild‐type, *Zmcaax‐1* and *Zmcaax‐2* plants following 300 mM mannitol treatment. Data are means ± SD of three biological replicates. Asterisks indicate significant differences: **P < 0.01, ***P < 0.001, as determined by Student's two‐tailed paired *t* tests. (h–j) qRT‐PCR analysis of alternative oxidase (Zm00001d002436) (h), peroxidase (Zm00001d014608) (i) and superoxide dismutase (Zm00001d036135) (j) in WT, *Zmcaax‐1* and *Zmcaax‐2* plants. Data are means ± SD of three biological replicates. Asterisks indicate significant differences: *P < 0.05, **P < 0.01, ***P < 0.001, as determined by Student's two‐tailed paired *t* tests.
**Figure S7** Disruption of *ZmCAAX* homologues in tobacco improves drought tolerance. (a) Multiple sequence alignment of the CAAX domain sequences from *Zea mays* and tobacco proteins. Conserved residues between maize and tobacco are highlighted in blue, while conserved residues within tobacco proteins are highlighted in purple. (b) qRT‐PCR analysis of *NtCAAX* expression levels in *NtCAAX‐RNAi* lines in tobacco. Data are means ± SD of three biological replicates. Asterisks indicate significant differences: ***P < 0.001, as determined by Student's two‐tailed paired *t* tests. (c) Phenotypic comparison of wild‐type and *NtCAAX‐RNAi* plants before and after 15 days of drought stress, followed by 3 days of rehydration. Scale bar = 10 cm. (d, e) Quantification of water loss rate (%) (d) and survival rate (%) after drought treatment (e) in wild‐type and *NtCAAX‐RNAi* plants. Data are means ± SD of three biological replicates. Asterisks indicate significant differences: *P < 0.05, **P < 0.01, ***P < 0.001, as determined by Student's two‐tailed paired *t* tests. (f–h) Determination of malondialdehyde levels (f), superoxide dismutase (g) and catalase activities (h) in wild‐type and *NtCAAX‐RNAi* plants before and after drought treatment. Data are means ± SD of three biological replicates. Asterisks indicate significant differences: *P < 0.05, **P < 0.01. ‘ns’ denotes no significant difference, as determined by Student's two‐tailed paired *t* tests.
**Figure S8** ZmCAAX cannot interact with the ZmNCED family member ZmVP14. (a) Phylogenetic tree analysis of *ZmNCED* family genes in maize. (b) Yeast two‐hybrid assay demonstrating that *ZmVP14* does not interact with *ZmCAAX*. Yeast cells harbouring the indicated plasmid combinations were grown on nonselective (SD/‐Trp/‐Leu) and selective (SD/‐Trp/‐Leu/‐His/‐Ade) media. Cells were diluted at three concentrations from left to right and photos were taken after 5 days.
**Figure S9** Determination of ZmNCED enzyme activity and ABA content in *ZmCAAX*‐mutant plants. (a, b) Relative expression of *ZmNCED* in wild‐type, *Zmcaax* mutants and *Zmcaax‐OE* plants analysed using qRT‐PCR. Data are means ± SD of three biological replicates. ‘ns’ indicates no significant difference. (c, d) Enzyme activity of ZmNCED in wild‐type, *Zmcaax* mutants and *ZmCAAX‐OE* plants. Data are means ± SD of three biological replicates. ‘ns’ indicates no significant difference. (e, f) Quantification of ABA content in wild‐type, *Zmcaax* mutants and *ZmCAAX‐OE* plants under control and drought stress conditions. Data are means ± SD of three biological replicates. Asterisks indicate significant differences: **P < 0.01, ***P < 0.001, ‘ns’ denotes no significant difference, as determined by Student's two‐tailed paired *t* tests.
**Figure S10** Characteristics of two *ZmCAAX*‐overexpressing maize lines. (a) Growth of transgenic recipient (B104) and *ZmCAAX‐OE* plants under the same environmental growing conditions. Scale bar = 10 cm. (b) Determination of chlorophyll a and chlorophyll b contents in B104 and *ZmCAAX*‐OE plants. Data are means ± SD of three biological replicates. Asterisks indicate significant differences: *P < 0.05, ‘ns’ indicates no significant difference. (c–e) Determination of zeaxanthin (c), α‐carotene (d) and β‐carotene (e) contents in B104 and *ZmCAAX*‐*OE* transgenic plants. Data are means ± SD of three biological replicates, with percentages indicating the increase relative to B104.
**Figure S11** Selection domestication analysis and genome‐wide association analysis of *ZmCAAX* and *ZmNCED3*. (a, b) Selection pressure analysis of ZmCAAX and ZmNCED3. Maize HapMap v3 SNP data indicate that nucleotide diversity in improved maize lines is significantly lower than that in teosinte. The red, green and blue lines represent nucleotide diversity in improved maize lines, landraces and teosinte, respectively. (c, d) Association of *ZmCAAX* and *ZmNCED3* gene region variants with post‐drought chlorophyll contents in over 220 sequenced maize genome‐wide association study materials.

**Table S1** Differentially expressed genes between *Zmcaax‐1* and wild‐type.

**Table S2** Differentially expressed genes between *Zmcaax‐1* and wild‐type under drought stress.

**Table S3** Primers used in this study.

**Text S1** Supplementary methods.

## Data Availability

The raw sequence data from this article can be found in The Maize Genetics and Genomics database (Maizegdb) (https://www.maizegdb.org/) under the following accession numbers: *ZmCAAX* (Zm00001d025860), *ZmNCED3* (Zm00001d041319). The raw RNA‐seq data from this study have been deposited in the National Genomics Data Center (NGDC) under accession number CRA022233 and are publicly available as of the date of publication.
