# Supplementary material for: Maize leaf yellowing gene ZmCAAX modulates growth and drought resistance by regulating abscisic acid contents through interaction with the ABA biosynthetic enzyme ZmNCED3
Source: Plant Biotechnol J. 2025 Jun 3;23(8):3431–50. doi: 10.1111/pbi.70147 (PMC12310864; doi:10.1111/pbi.70147)
Supplement: Supplementary file 1 — Figure S1 Phenotype characterization of the yp1 mutants. (a) Wild‐type (RP125) and yp1 mutants at four developmental stages, photographed at various time points post‐germination. (b) Quantification of pigment contents in the second leaf of wild‐type and yp1 mutants, including Chlorophyll a (Chl a), chlorophyll b (Chl b), carotenoid (Car). Data are means ± SD of three biological replicates. Asterisks indicate significant differences: *P < 0.05, **P < 0.01, ***P < 0.001, ns indicates no significant difference P ≥ 0.05. (c, d) Comparative analysis of plant height (c) and flowering time (d) in wild‐type and yp1 mutants plants. Data are means ± SD of three biological replicates. Asterisks indicate significant differences: **P < 0.01, ns indicates no significant difference P ≥ 0.05. Figure S2 Abnormal chloroplast development in the yp1 mutants. (a) Quantification of epidermal structures, including macrohairs (mh), prickle hairs (ph), bicellular hairs (bh) and stomatal complexes (st), per 2.1 mm2 of the adaxial leaf surface in wild‐type and yp1 mutants plants. Data are means ± SD (n = 10). Significant differences were determined by two‐way ANOVA. Asterisks indicate significant differences: *P < 0.05, **P < 0.01, ***P < 0.001. (b) Immunoblot analysis of key photosynthetic proteins in wild‐type and yp1 mutants plants. RbcL (Rubisco large subunit), AtpB (ATP synthase β‐subunit) and PsbD (PSII reaction centre protein D2) were detected using specific antibodies. Serial dilutions (1, 0.5, 0.1) of total protein extracts were loaded for semi‐quantitative comparison. Ponceau S staining was used as a loading control. (c) qRT‐PCR analysis of photosynthetic complex subunit expression levels in wild‐type and yp1 mutants plants. Data are presented as means ± SD of three biological replicates. Asterisks denote statistically significant differences: *P < 0.05, **P < 0.01, ***P < 0.001, as determined by Student's two‐tailed paired t tests. Figure S3 Genetic and phenotypic characterization [file PBI-23-3431-s001.pdf]

# Figure S1.

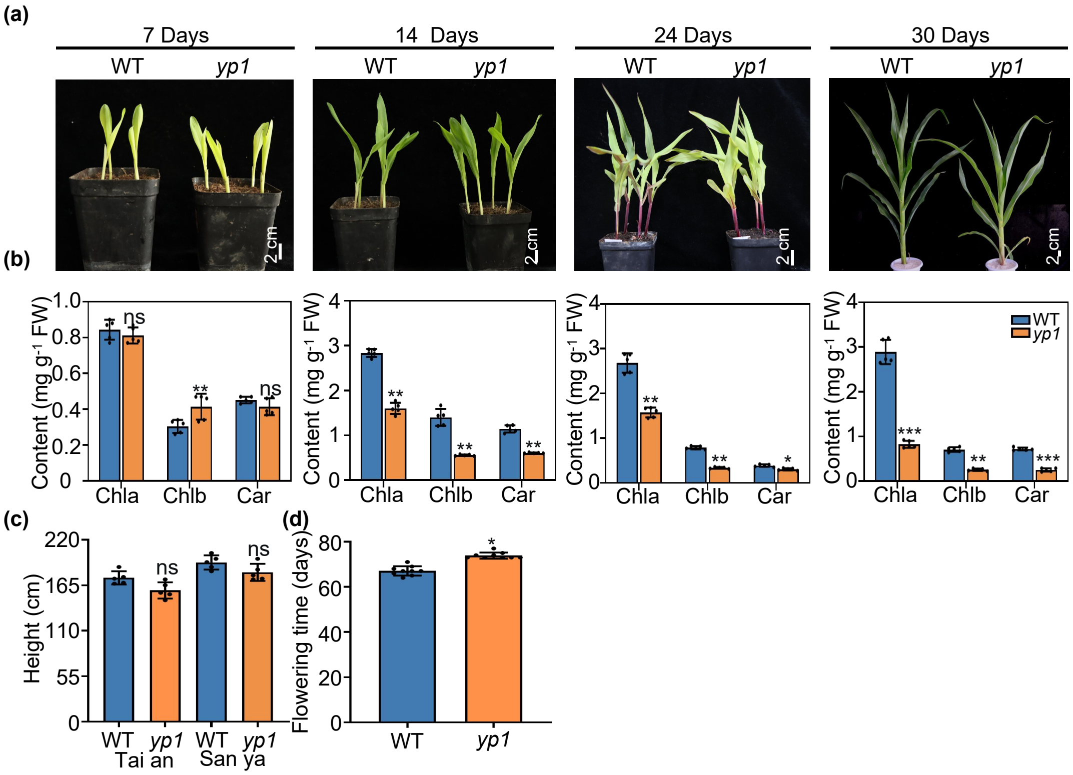


**Figure S1 Phenotype characterization of the *yp1* mutants.**

(a) Wild-type (RP125) and *yp1* mutants at four developmental stages, photographed at various time points post-germination.

(b) Quantification of pigment contents in the second leaf of wild-type and *yp1* mutants, including Chlorophyll a (Chl a), chlorophyll b (Chl b), carotenoid (Car). Data are means ± SD of three biological replicates. Asterisks indicate significant differences: $*P < 0.05$, $**P < 0.01$, $***P < 0.001$, ns indicates no significant difference $P \geq 0.05$.

(c, d) Comparative analysis of plant height (c) and flowering time (d) in wild-type and *yp1* mutants plants. Data are means ± SD of three biological replicates. Asterisks indicate significant differences: $**P < 0.01$, ns indicates no significant difference $P \geq 0.05$.

# Figure S2.

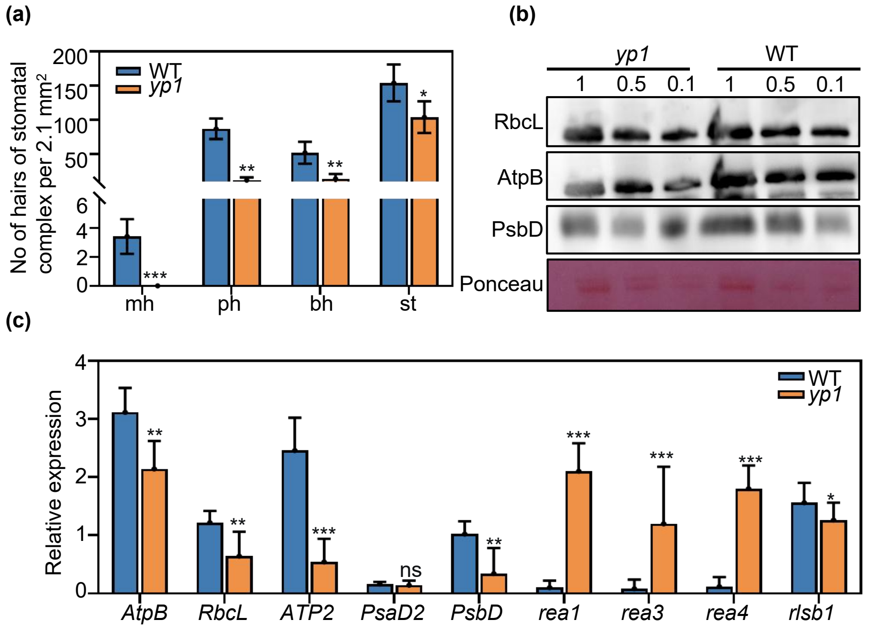


**Figure S2 Abnormal chloroplast development in the *yp1* mutants.**

(a) Quantification of epidermal structures, including macrohairs (mh), prickle hairs (ph), bicellular hairs (bh), and stomatal complexes (st), per 2.1 mm² of the adaxial leaf surface in wild-type and *yp1* mutants plants. Data are means ± SD (n = 10). Significant differences were determined by two-way ANOVA. Asterisks indicate significant differences: *$P < 0.05$, **$P < 0.01$, ***$P < 0.001$.

(b) Immunoblot analysis of key photosynthetic proteins in wild-type and *yp1* mutants plants. RbcL (Rubisco large subunit), AtpB (ATP synthase β-subunit), and PsbD (PSII reaction center protein D2) were detected using specific antibodies. Serial dilutions (1, 0.5, 0.1) of total protein extracts were loaded for semi-quantitative comparison. Ponceau S staining was used as a loading control.

(c) qRT-PCR analysis of photosynthetic complex subunit expression levels in wild-type and *yp1* mutants plants. Data are presented as means ± SD of three biological replicates. Asterisks denote statistically significant differences: *$P < 0.05$, **$P < 0.01$, ***$P < 0.001$, as determined by student's two-tailed paired *t* tests.

# Figure S3.

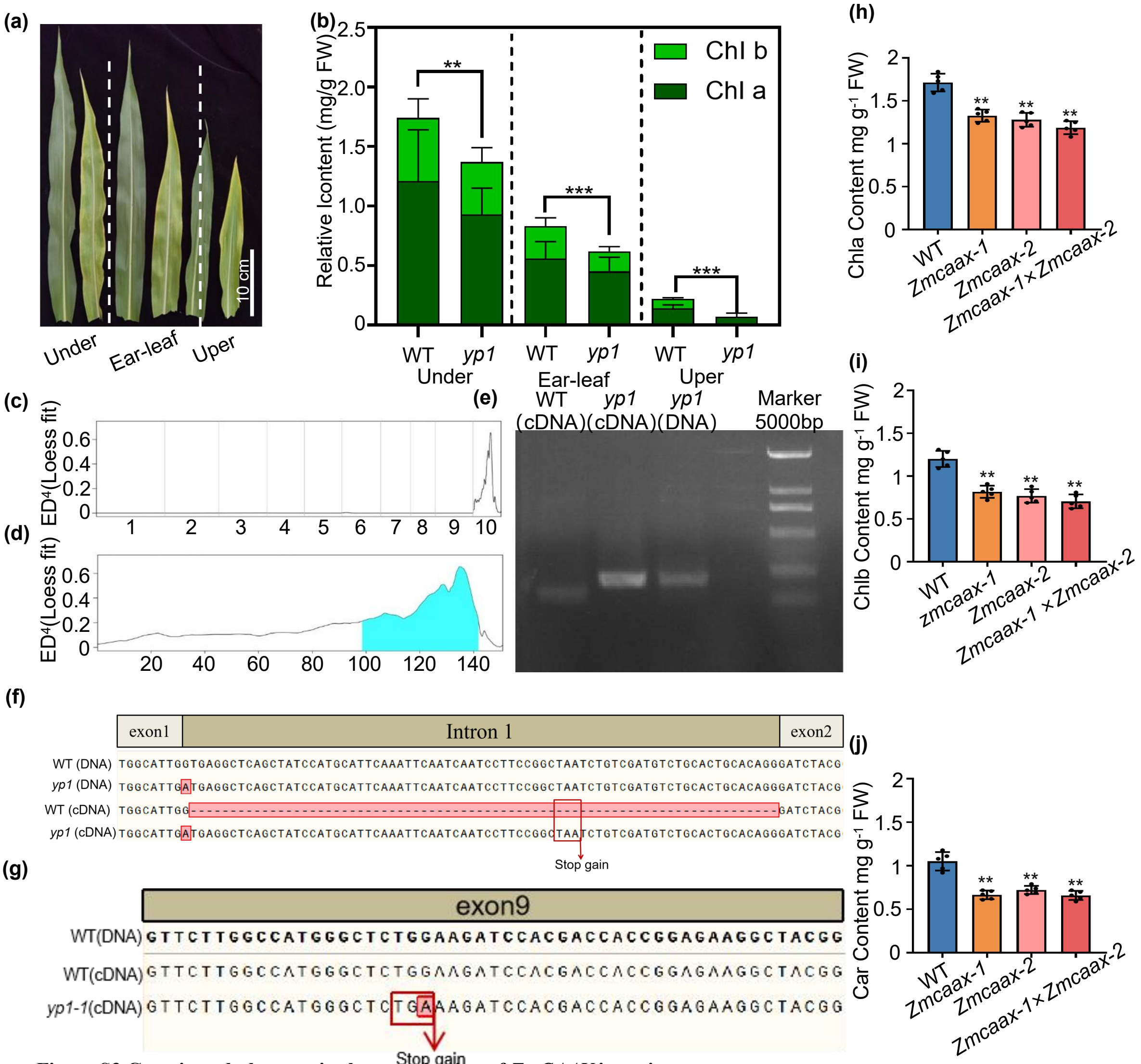


**Figure S3 Genetic and phenotypic characterization of *ZmCAAX* in maize.**

(a, b) Phenotypic characterization of leaves at different positions: lower leaves (below the ear), ear leaves (at the ear position), and upper leaves (above the ear). Scale bar = 10 cm. (b) Chlorophyll a and chlorophyll b contents were quantified in leaves from the B73 × *yp1* $F_2$ segregating population. Data are means ± SD of three biological replicates. Asterisks indicate significant differences: **$P < 0.01$, ***$P < 0.001$, as determined by student's two-tailed paired *t* tests.

(c, d) Bulked segregant analysis (BSA) sequencing profiles showing the distribution of genetic variations associated with the *yp1* mutant phenotype.

(e–g) *ZmCAAX* allele identification and sequencing results analysis.

(h–j) Quantification of chlorophyll a (h), chlorophyll b (i), and total carotenoid (j) contents in wild-type, *Zmcaax-1*, *Zmcaax-2*, and *Zmcaax-1*×*Zmcaax-2* mutants. Data are means ± SD of three biological replicates. Asterisks indicate significant differences: **$P < 0.01$, as determined by student's two-tailed paired *t* tests.

# Figure S4.

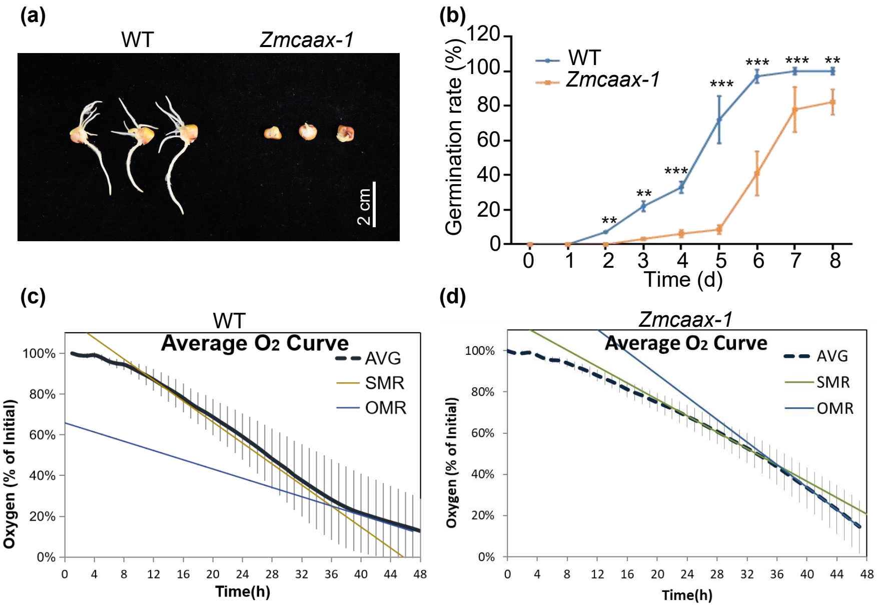


**Figure S4 *Zmcaax* mutation inhibits seed germination.**

(a) Comparison of seed germination between wild-type and *Zmcaax-1* mutant seeds. Scale bar = 1 cm.

(b) Germination rate over time in wild-type and *Zmcaax-1* mutant seeds. Data represent means ± SEM, as determined by Student's *t*-test. **$P$<0.01, ***$P$<0.001.

(c, d) Oxygen consumption dynamics during germination in wild-type and *Zmcaax* mutant seeds.

# Figure S5.

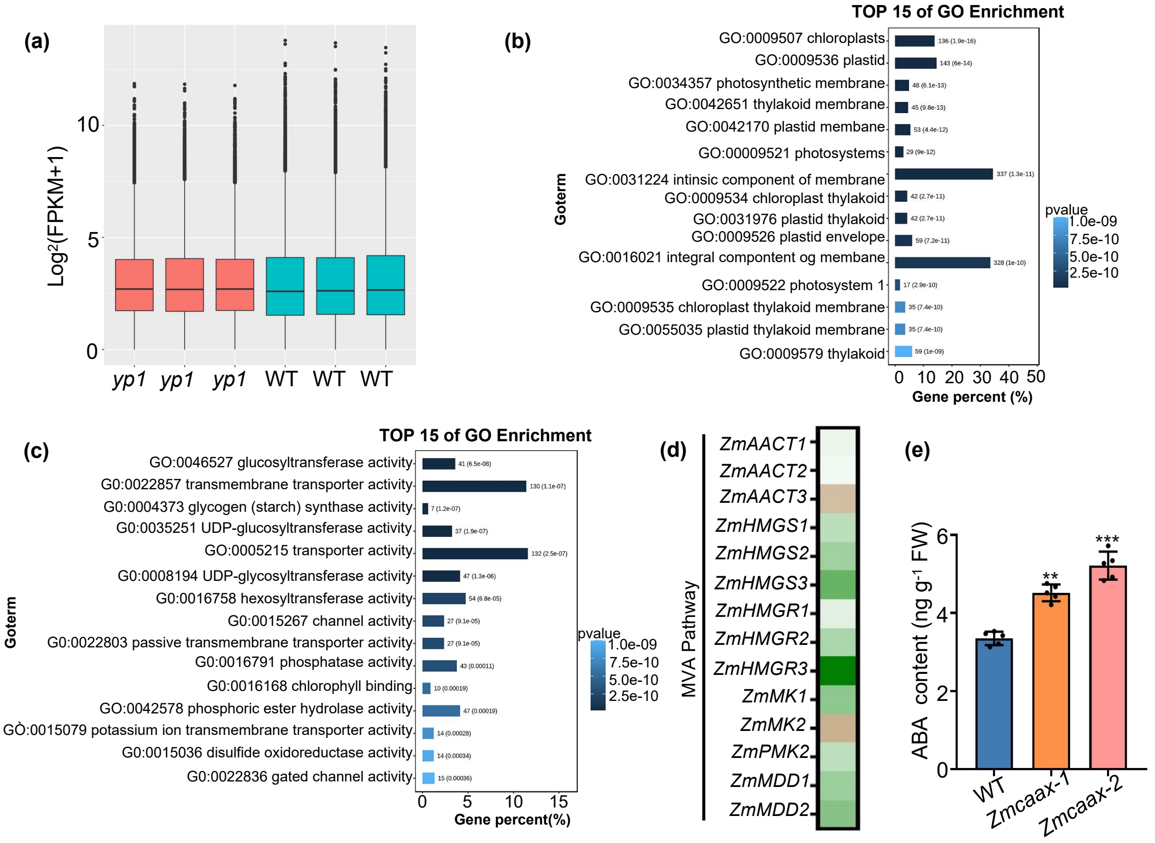


**Figure S5 Transcriptomic analysis and ABA quantification in *Zmcaax* mutants.**

(a) Principal component analysis (PCA) of transcriptomic data showing clustering of biological replicates for wild-type and *Zmcaax* mutant plants.

(b, c) GO functional enrichment analysis of genes involved in pathways related to molecular functions and cellular localization. Numbers represent the number of enriched genes.

(d) Heatmap of MVA pathway gene expression in wild-type and *Zmcaax* mutant plants, based on RNA-seq data.

(e) Determination of ABA content in wild-type and *Zmcaax* mutant plants. Data are means ± SD of three biological replicates. Asterisks indicate significant differences: $^{**}P < 0.01$, $^{***}P < 0.001$.

# Figure S6.

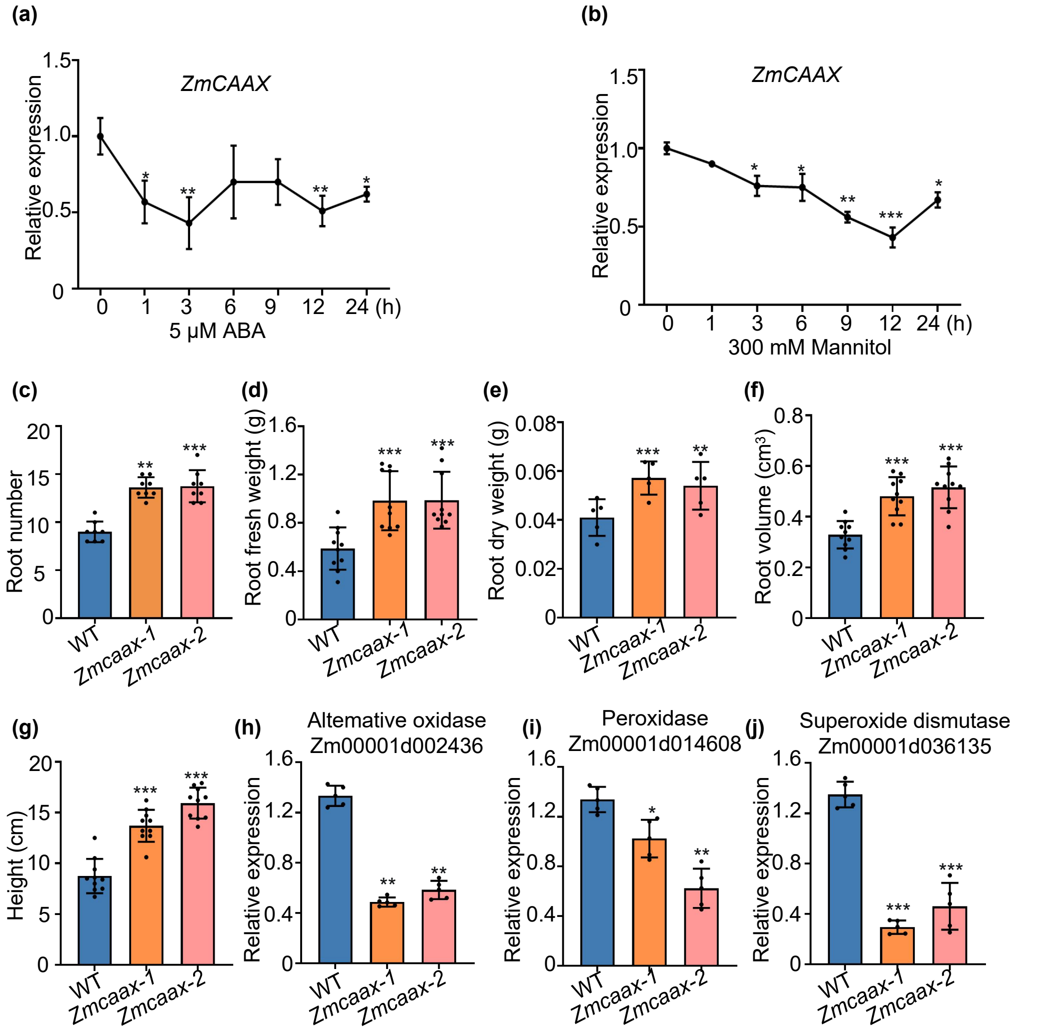


**Figure S6 *ZmCAAX* genes participate in maize drought tolerance.**

(a, b) qRT-PCR analysis of *ZmCAAX* expression levels in response to 5 μM ABA (a) and 300 mM mannitol (b) treatment. Data are means ± SD of three biological replicates.

(c–g) Morphological analysis of *Zmcaax-1* and *Zmcaax-2* mutants seedlings under drought stress. Root number (c), root fresh weight (d), root dry weight (e), root volume (f), and seedling height (g) were quantified in wild-type, *Zmcaax-1*, and *Zmcaax-2* plants following 300 mM mannitol treatment. Data are means ± SD of three biological replicates. Asterisks indicate significant differences: $**P < 0.01$, $***P < 0.001$, as determined by student's two-tailed paired *t* tests.

(h–j) qRT-PCR analysis of alternative oxidase (Zm00001d002436) (h), peroxidase (Zm00001d014608) (i), and superoxide dismutase (Zm00001d036135) (j) in WT, *Zmcaax-1*, and *Zmcaax-2* plants. Data are means ± SD of three biological replicates. Asterisks indicate significant differences: $*P < 0.05$, $**P < 0.01$, $***P < 0.001$, as determined by student's two-tailed paired *t* tests.

# Figure S7.

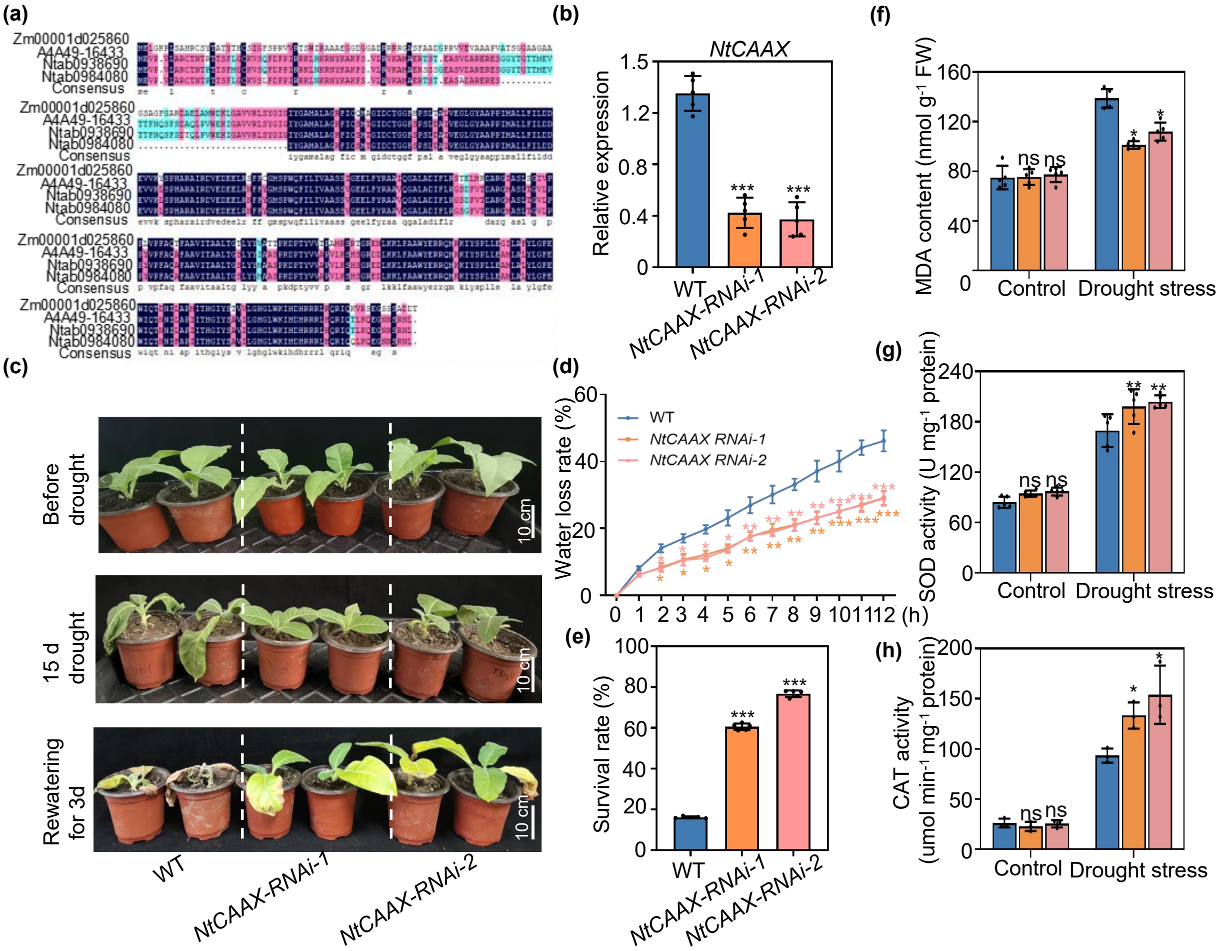


**Figure S7 Disruption of *ZmCAAX* homologs in tobacco improves drought tolerance.**

(a) Multiple sequence alignment of the CAAX domain sequences from Zea mays and tobacco proteins. Conserved residues between maize and tobacco are highlighted in blue, while conserved residues within tobacco proteins are highlighted in purple.

(b) qRT-PCR analysis of *NtCAAX* expression levels in *NtCAAX-RNAi* lines in tobacco. Data are means ± SD of three biological replicates. Asterisks indicate significant differences: ****P* < 0.001, as determined by student's two-tailed paired *t* tests.

(c) Phenotypic comparison of wild-type and *NtCAAX-RNAi* plants before and after 15 days of drought stress, followed by 3 days of rehydration. Scale bar = 10 cm.

(d, e) Quantification of water loss rate (%) (d) and survival rate (%) after drought treatment (e) in wild-type and *NtCAAX-RNAi* plants. Data are means ± SD of three biological replicates. Asterisks indicate significant differences: **P* < 0.05, ***P* < 0.01, ****P* < 0.001, as determined by student's two-tailed paired *t* tests.

(f–h) Determination of malondialdehyde levels (f), superoxide dismutase (g) and catalase activities (h) in wild-type and *NtCAAX-RNAi* plants before and after drought treatment. Data are means ± SD of three biological replicates. Asterisks indicate significant differences: **P* < 0.05, ***P* < 0.01. "ns" denotes no significant difference, as determined by student's two-tailed paired *t* tests.

# Figure S8.

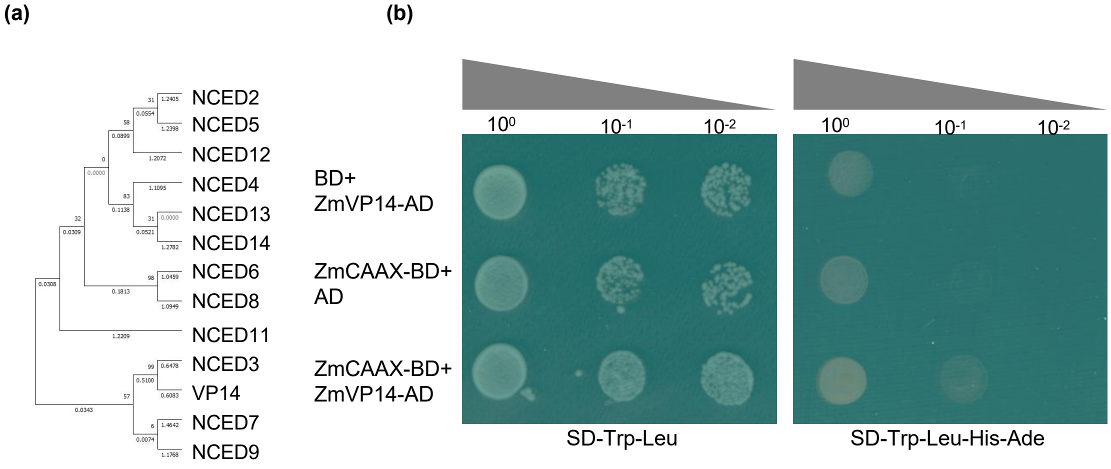


**Figure S8 ZmCAAX cannot interact with the ZmNCED family member ZmVP14.**

(a) Phylogenetic tree analysis of *ZmNCED* family genes in maize.

(b) Yeast two-hybrid assay demonstrating that *ZmVP14* does not interact with *ZmCAAX*. Yeast cells harboring the indicated plasmid combinations were grown on nonselective (SD/-Trp/-Leu) and selective (SD/-Trp/-Leu/-His/-Ade) media. Cells were diluted at three concentrations from left to right, and photos were taken after 5 days.

# Figure S9.

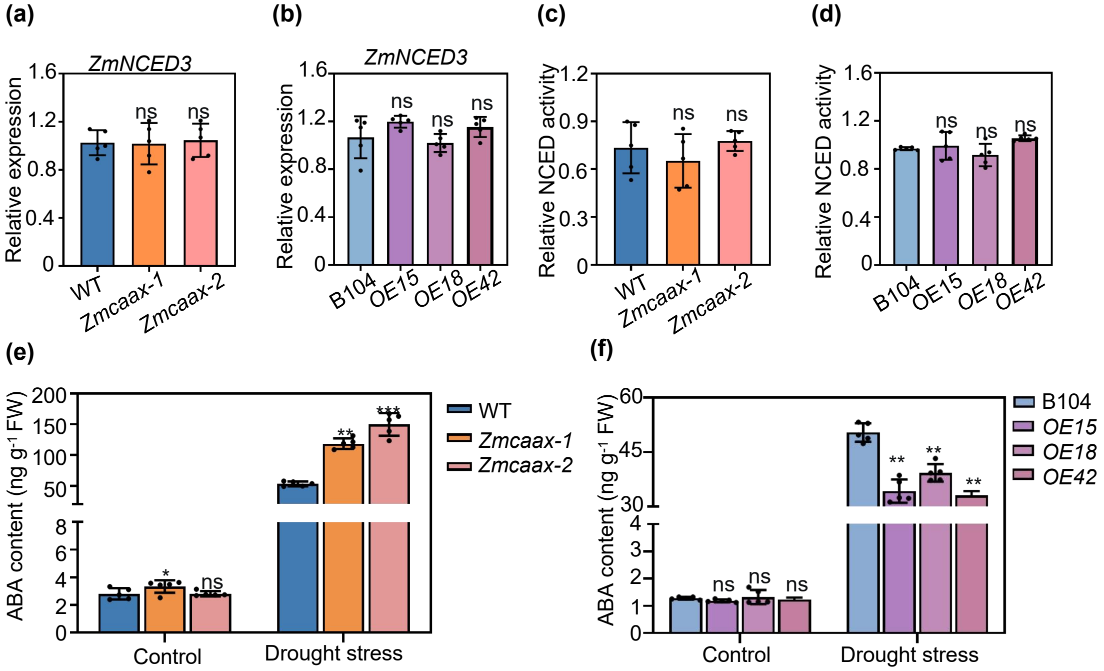


**Figure S9 Determination of ZmNCED enzyme activity and ABA content in *ZmCAAX*-mutant plants.**

(a, b) Relative expression of *ZmNCED* in wild-type, *Zmcaax* mutants, and *Zmcaax-OE* plants analyzed using qRT-PCR. Data are means ± SD of three biological replicates. "ns" indicates no significant difference.

(c, d) Enzyme activity of ZmNCED in wild-type, *Zmcaax* mutants, and *ZmCAAX-OE* plants. Data are means ± SD of three biological replicates. "ns" indicates no significant difference.

(e, f) Quantification of ABA content in wild-type, *Zmcaax* mutants, and *ZmCAAX-OE* plants under control and drought stress conditions. Data are means ± SD of three biological replicates. Asterisks indicate significant differences: **$P <$ 0.01, ***$P <$ 0.001, "ns" denotes no significant difference, as determined by student's two-tailed paired $t$ tests.

# Figure S10.

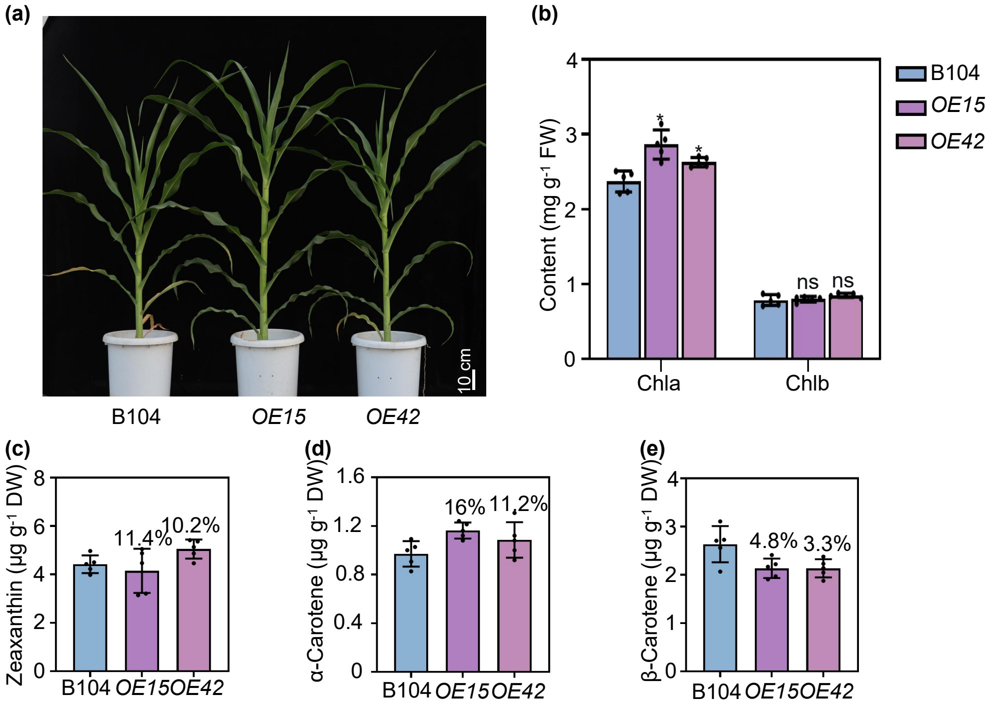


**Figure S10 Characteristics of two *ZmCAAX*-overexpressing maize lines.**

(a) Growth of transgenic recipient (B104) and *ZmCAAX-OE* plants under the same environmental growing conditions. scale bar = 10 cm.

(b) Determination of chlorophyll a and chlorophyll b contents in B104 and *ZmCAAX*-OE plants. Data are means ± SD of three biological replicates. Asterisks indicate significant differences: $*P < 0.05$, "ns" indicates no significant difference.

(c–e) Determination of zeaxanthin (c), α-carotene (d), and β-carotene (e) contents in B104 and *ZmCAAX-OE* transgenic plants. Data are means ± SD of three biological replicates, with percentages indicating the increase relative to B104.

# Figure S11.

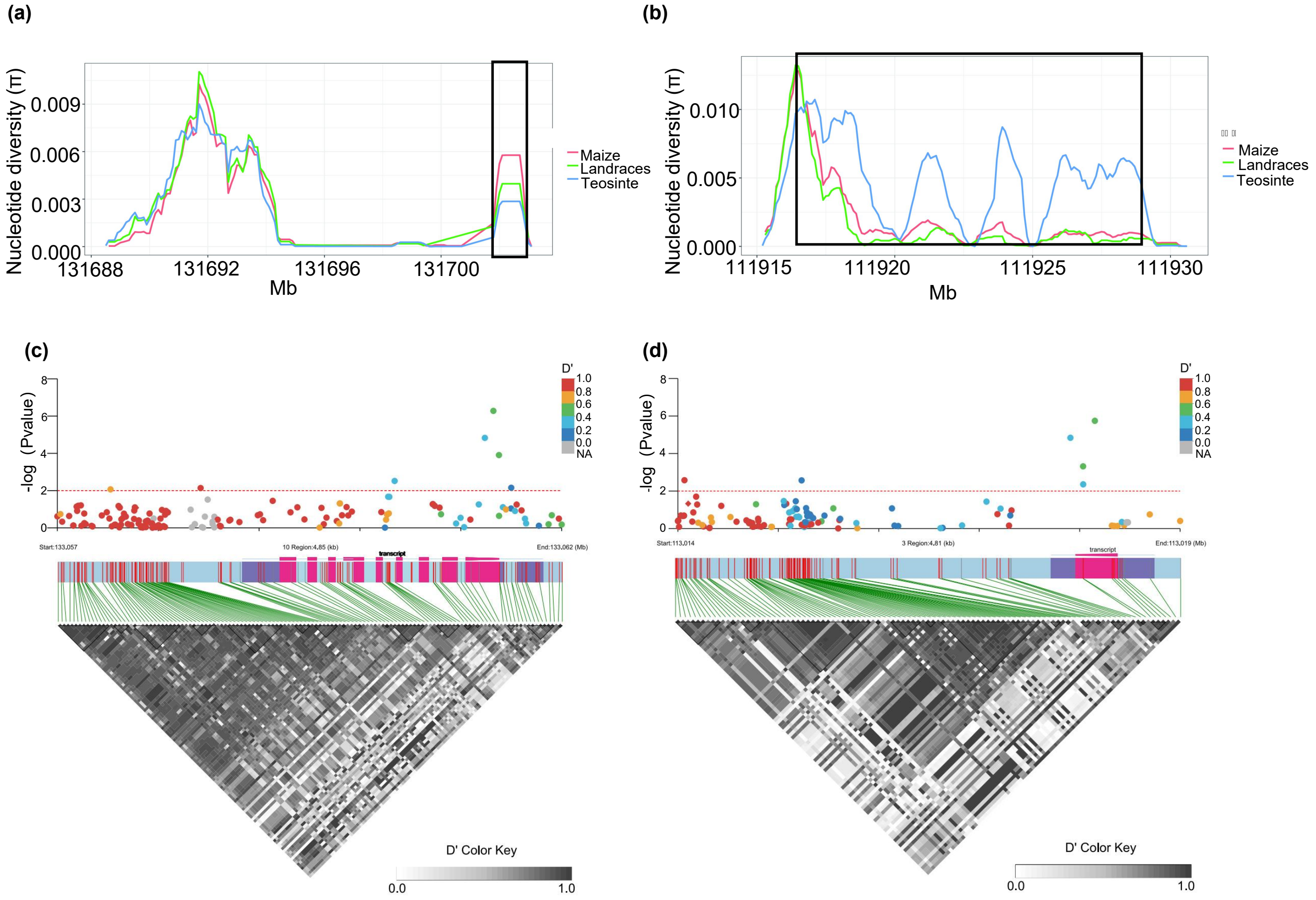


**Figure S11 Selection domestication analysis and genome-wide association analysis of *ZmCAAX* and *ZmNCED3*.**

(a, b) Selection pressure analysis of ZmCAAX and ZmNCED3. Maize HapMap v3 SNP data indicate that nucleotide diversity in improved maize lines is significantly lower than that in teosinte. The red, green, and blue lines represent nucleotide diversity in improved maize lines, landraces, and teosinte, respectively.

(c, d) Association of *ZmCAAX* and *ZmNCED3* gene region variants with post-drought chlorophyll contents in over 220 sequenced maize genome-wide association study materials.
