# Supplementary material for: Maize leaf yellowing gene ZmCAAX modulates growth and drought resistance by regulating abscisic acid contents through interaction with the ABA biosynthetic enzyme ZmNCED3
Source: Plant Biotechnol J. 2025 Jun 3;23(8):3431–50. doi: 10.1111/pbi.70147 (PMC12310864; doi:10.1111/pbi.70147)
Supplement: Supplementary file 5 — Text S1 Supplementary methods. [file PBI-23-3431-s004.docx]

**Hormone content determination**

To measure the hormone content, we grew wild-type, *Zmcaax* mutants, B104, and *ZmCAAX*-OE plants. Fresh plant materials were freeze-dried in liquid nitrogen and stored at −80°C until further analysis. The dried plant material was then homogenized and ground into a fine powder using a grinder. The dried powder sample (weighing 100 mg) was extracted with 1.5 mL of a solution composed of MeO:H₂O (79.9:20:0.1). The extract was vortexed and sonicated for 30 min, followed by incubation at 4°C for 12 h. After centrifugation, the supernatant was collected. The residue was re-extracted with 1 mL of MeOH, sonicated for 30 min, and centrifuged again. The supernatants were combined, evaporated to dryness under a nitrogen stream, and reconstituted in 100 μL of a MeO:H₂O (50:50) solution. The solution was filtered through a 0.22-μm filter for further LC–MS analysis. The samples were analyzed using a high-performance liquid chromatography-tandem mass spectrometry (HPLC-MS/MS system; Triple Quadrupole 4500). The HPLC conditions were as follows: the column was a HYPERSIL GOLD C18 column (3 μm, 2.1 mm × 100 mm); solvent A was H₂O with 0.1% FA, and solvent B was MeOH. The gradient program was set as follows: 90% A from 0 to 0.2 min, 90% A maintained until 8 min, followed by 10% A at 8.1 min and maintained until 10 min. The flow rate was set to 0.3 mL/min, the column temperature was 35°C, and the injection volume was 5 μL. The Triple Quadrupole 4500 HPLC-MS/MS system, equipped with an ESI ion source, was operated in both positive and negative ion modes, controlled by Analyst 1.6.3 software (Sciex). The ESI source operation parameters were as follows: ion source, ESI+/-; ion spray voltage, 5500V/4500V; source temperature, 550°C; curtain gas pressure, 30 psi; and collision gas pressure, 9 psi. DP and CE were optimized for individual MRM transitions. A specific set of MRM transitions were monitored for each period based on the phytohormones eluted during that time.

**RNA–seq analysis**

For RNA-seq analysis, wild-type and *Zmcaax* mutant plants were sampled at various time points with or without 300 mM mannitol treatment. Three biological replicates were performed for each time point under normal conditions and at 6 h after 300 mM mannitol treatment. Sequencing libraries were prepared using the Illumina TruSeq RNA sample prep kit and sequenced on the Illumina HiSeq X Ten System. The quality of raw sequence reads was assessed using Fastp v. 0.12.4 (Chen et al., 2018).

**Scanning electron microscopy observation**

SEM was used to analyze the morphological characteristics of epidermal hairs on the leaves of wild type and Zmcaax mutants as well as stomatal opening and closing before and after drought treatment. Fresh ear leaves from the wild-type and mutant plants were cut into 0.5-cm sections and fixed in 2.5% glutaraldehyde solution at 4°C for 24 h. The samples were then washed five times with phosphate buffer (pH 7.2) for 10 min each. After washing, the samples were dehydrated in a graded ethanol series (25%, 50%, 75%, 95%, and 100%) and critical point dried with CO₂. Subsequently, the samples were examined using a JEOL scanning electron microscope (Hitachi, Kyoto, Japan). For each sample, five leaf area images were used for statistical analysis. Stomatal aperture was measured by averaging the values from at least 100 stomata across five biological replicates.

**Yeast two-hybrid analysis**

To examine ZmCAAX interact with ZmNCEDs, the full-length sequence fragment of *ZmCAAX* was cloned into the pGBKT7 vector digested with *Nde*I and *Eco*RI to generate the construct ZmCAAX-BD. Similarly, the full-length coding sequence of ZmNCEDs were cloned into the pGADT7 vector to generate the ZmNCEDs-AD construct. These expression constructs were cotransformed into yeast Y2H gold strain cells. The transformed cells were selected on the SD-Leu-Trp medium and further validated on SD-Leu-Trp-His-Ade medium.

**Firefly luciferase complementation imaging (LCI) assays**

The full-length CDS of *ZmNCED3* was cloned into the pCAMBIA1300-cLUC (cLUC) vector to generate the *ZmNCED3-cLUC* construct, whereas the full-length CDS of *ZmCAAX* was cloned into the pCAMBIA1300-nLUC (nLUC) vector to generate the *nLUC-ZmCAAX* construct. Next, 1 mL of *A. tumefaciens* cells harboring *nLUC-ZmCAAX* and *ZmNCED3-cLUC* were mixed to obtain the following combinations. Each combination of *A. tumefaciens* cells was infiltrated separately into *N. benthamiana* leaves and allowed to express for 48 h. Luciferase signals were detected using a CCD camera (Olympus BX51). The LUC and REN luciferase activities in *N. benthamiana* were measured using the dual-luciferase reporter assay system and a multimode reader (Vazyme, Nanjing, China). The ratio of LUC to REN activities was calculated to determine the relative LUC activity.

**Bimolecular fluorescence complementation (BiFC) analysis**

The full-length coding sequences of *ZmCAAX* and *ZmNCED3* were amplified and cloned into the BiFC vector pSET-n/cYFP. The resulting constructs *ZmNCED3-cYFP/nYFP-ZmCAAX*, *ZmNCED3-cYFP/nYFP-ZmCAAX*, *cYFP/nYFP-ZmCAAX*, and *ZmNCED3-cYFP/nYFP* were transformed into *N. benthamiana* leaves and expressed for 56 h. Fluorescence was then detected using confocal microscopy (Zeiss, Carl Zeiss, Gottingen, Germany). BiFC assays were performed as previously described (Zhang et al., 2021).

**Co-immunoprecipitation (Co-IP) assays**

For Co-IP experiments, the plasmid pairs 35S::*ZmCAAX*-GFP/35S::*ZmNCED3*-3HA and *35S::GFP*/35S::*ZmNCED3*-3HA were co-infiltrated into *N. benthamiana* leaves. Approximately 72 h after infiltration, leaves were collected and homogenized in a protein extraction buffer (50 mM Tris-MES, pH 8.0; 0.5 M sucrose; 1 mM MgCl₂; 10 mM EDTA; 5 mM DTT; and 1 mM phenylmethylsulfonyl fluoride). Following protein extraction, anti-GFP antibodies (Transgene, Beijing, China; 1:300 dilution) coupled to magnetic beads were mixed with the protein samples and incubated at 4°C for 5–6 h. The captured proteins were separated through sodium dodecyl sulfate-polyacrylamide gel electrophoresis (SDS-PAGE), and *ZmCAAX* and *ZmNCED3* were detected using anti-GFP (1:1,000 dilution) and anti-HA (Abcam, Shanghai, China; 1:1,000 dilution) antibodies, respectively.
